# New Insights into the Hydrogen Evolution Mechanism near the Ni/YSZ Triple Phase Boundary during Steam Electrolysis: A Patterned Model Electrode Study

**DOI:** 10.1021/acselectrochem.4c00031

**Published:** 2024-11-04

**Authors:** Christoph W. Thurner, Kevin Ploner, Daniel Werner, Simon Penner, Engelbert Portenkirchner, Bernhard Klötzer

**Affiliations:** † Institute of Physical Chemistry, University of Innsbruck, Innrain 52c, A-6020 Innsbruck, Austria; ‡ Plansee SE, Metallwerk-Plansee-Strasse 71, 6600 Reutte, Austria; § Ceratizit S.A., 101 Rue de Holzem, 8232 Mamer, Luxembourg

**Keywords:** near-ambient pressure XPS, yttria-stabilized zirconia, solid oxide electrolysis cell, electrochemical impedance spectroscopy, e-field modeling, patterned model electrode

## Abstract

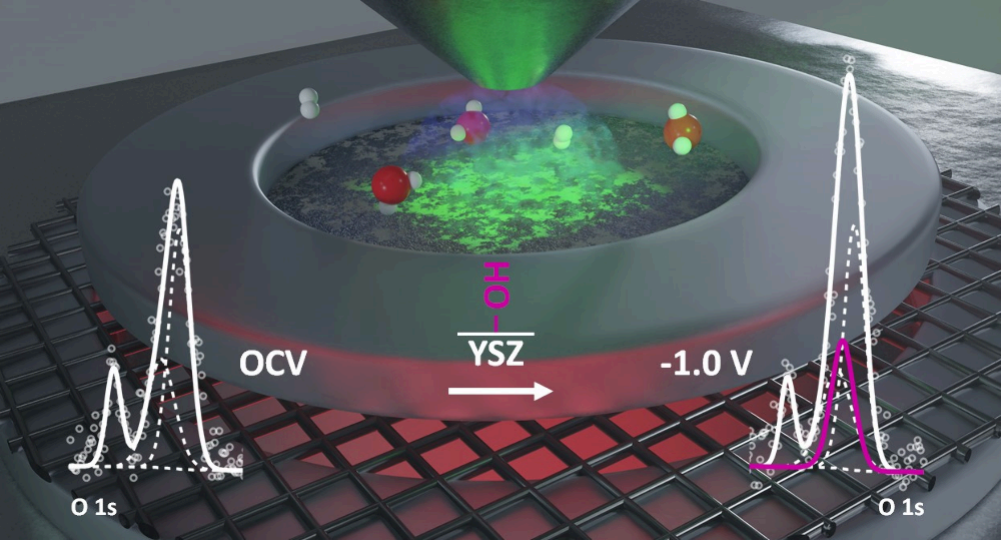

Solid oxide cell technologies play a crucial role in climate change mitigation by enabling the reversible storage of renewable energy. Understanding the electrochemical high-temperature reaction mechanisms and the catalytic role of the electrode and electrolyte materials is essential for advancing power-to-H_2_ technologies. Despite its significance, limited *in situ* spectroscopic research focusing on nickel and yttria-stabilized zirconia (Ni/YSZ) is available. We employ near-ambient pressure X-ray photoelectron spectroscopy (NAP-XPS) to investigate 2D porous Ni/YSZ model electrodes with variable YSZ domain sizes and triple phase boundary (TPB) lengths. Focusing on the hydrogen evolution reaction (HER), we provide a mechanistic explanation for why surface hydroxylation and electrochemical activity are correlated with the YSZ surface area and YSZ domain size and unravel the specific mechanistic role of the YSZ surface. A direct comparison of normalization of the measured total electrolysis current to the purely geometrical length of the TPB vs an electrified “catchment area” next to the TPB, exhibiting strong enough electric fields, is the key to a correct quantitative description of the individual elementary steps of water electrolysis on Ni/YSZ. By combining electrochemical impedance spectroscopy, NAP-XPS, and electric field modeling, the local water reduction process near the TPB can be described, indicating optimized structural parameters for improved HER performance.

## Introduction

Within climate change mitigation strategies, the reversible storage and release of renewable energy by solid oxide cell (SOC) technologies is of paramount importance to society.^1,2^ In particular, within power-to-H_2_ technologies, a comprehensive understanding of the high-temperature hydrogen evolution reaction (HER) mechanism and the associated electrocatalytic properties of the metal–electrolyte interface is essential for driving knowledge-based innovations.^3^ Molecular H_2_ plays a fundamental role in the utilization strategies of CO_2_ for producing e-fuels and chemicals.^1,4^ However, despite its significance, there is a scarcity of *in situ* studies focusing on the most widely applied metal–ceramic (cermet) electrode material, which comprises a porous but percolated structure of both nickel and yttria-stabilized zirconia (YSZ) grains.^5^ Existing studies often revolve around investigating poisoning agents using Raman spectroscopy^6−8^ or are oriented in the empirical and theoretical improvement of the 3D cermet micro- and mesostructures based on exchange-current optimization restricted to fuel cell mode only.^1,9−11^

While *in situ* studies applying near-ambient pressure X-ray photoelectron spectroscopy (NAP-XPS) on H_2_-exchanging electrodes exist, these are rather centered on perovskite- or other mixed conducting oxide-based electrodes^12,13^ or Pt/YSZ interfaces.^14^ Bluhm et al. delved into the Ni/YSZ interface and scrutinized the anodic oxidation of H_2_.^15^ Mechanistic predictions regarding the water reduction reaction (eq 1) over Ni/YSZ interfaces rely primarily on theoretical calculations^16,17^ and electrochemical polarization characteristics, including DC and AC methods.^18,19^
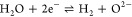
1

To experimentally elucidate the specific reaction mechanism occurring over Ni/YSZ electrodes during the HER, our focus lies on investigating specially designed model thin-film cells^20^ via *in situ* NAP-XPS. This approach allows for a detailed examination of the chemical surface composition, oxidation states, and adsorbate chemistry of the electrochemically active zone of the working electrode (WE).^21−23^ Recently, we demonstrated that a 2D network of a metallic, electron-conducting thin film deposited on a YSZ solid electrolyte enables the investigation of temperature- or potential-induced surface changes near the electrochemically active triple phase boundary (TPB).^20^

This study aims to comparatively investigate the structure–activity relationship of two specifically designed Ni/8-YSZ 2D-structured thin-film model electrodes, differing in their geometric TPB properties (i.e., TPB length and YSZ domain size). By electrifying the TPB at three different temperatures (400, 450, and 500 °C) in a H_2_:H_2_O 1:9 gas mixture (1 mbar), we successfully identified the structure- and polarization-dependent appearance of surface hydroxyl species *in situ*, providing the first pieces of the mechanistic puzzle. The choice of operating temperatures below the technological values around 800 °C allows for the enrichment of the intermediate YSZ-bonded hydroxyl species to NAP-XPS-detectable levels. By correlating the surface response upon polarization with the electrochemical cell characteristics monitored by electrochemical impedance spectroscopy (EIS) while simultaneously recording NAP-XPS spectra, we could further refine the mechanistic picture. Additionally, a local electric field (e-field) modeling approach provides insights into the dependence of electrochemical activity on the specific near-TPB structures.

Eventually, the integration of *ex situ* structural and microscopic characterization, NAP-XPS and EIS experiments, and theoretical electric field modeling allows us to deduce previously unknown details of the reaction mechanism of water vapor electrolysis on Ni/YSZ electrodes. This paves the way for knowledge-based improvements in cell performance by optimizing the YSZ surface domain size and, consequently, the local electric field properties at the TPB.

## Experimental Details

### Cell Preparation

The solid electrolyte utilized in this study is a commercially fabricated button cell (Ø 5 mm, Kerafol, Germany) consisting of a 150 μm thick 8 mol % yttria-stabilized zirconia ((ZrO_2_)_0.92_(Y_2_O_3_)_0.08_, 8-YSZ) substrate and a 10 μm thick adhesive top layer of 10 mol % gadolinium-doped ceria (Gd_0.1_Ce_0.9_O_1.95_, GDC-10) for the Pt/GDC-based counter electrode (CE) preparation.^20^ The specialized design of the CE includes a highly active porous Pt/GDC-10 layer with an optimized number of TPB sites and a pure Pt current collector layer. Previous studies have shown that overpotential contributions dedicated to CE processes can be neglected for such a CE setup.^24−26^ Nonetheless, this study examined the CE overpotential contributions through a symmetrical cell approach in order to correct the Ni/8-YSZ EIS data.

**Figure 1:**
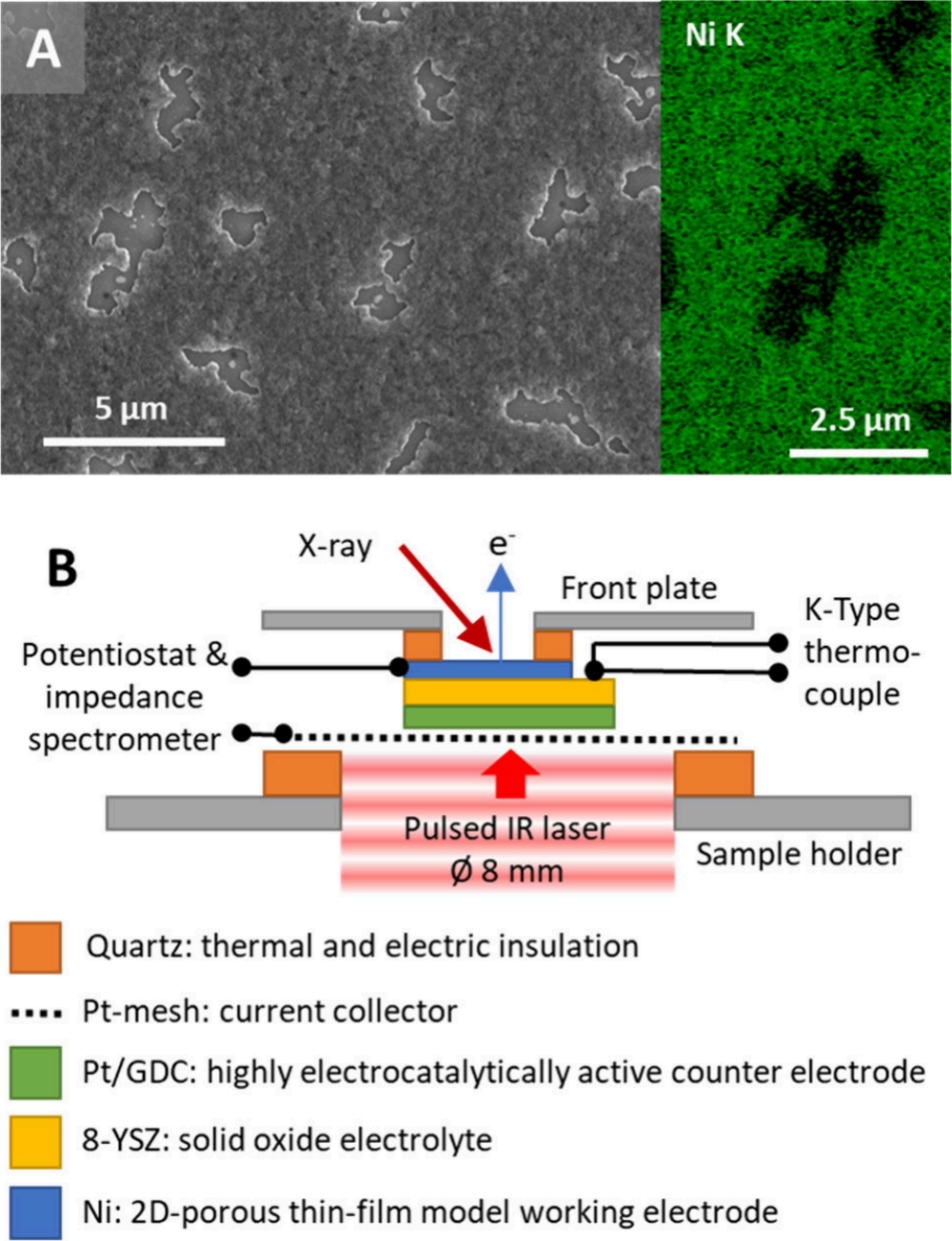
(A) SEM image depicting the 2D porous thin-film model electrode, showcasing dewetted YSZ domains. The mapped Ni K intensity (inset) corroborates the holey and percolated Ni structure of the thin film. (B) Cross-sectional schematic illustrating the entire solid oxide electrochemical cell setup mounted on the sample holder in the NAP-XPS chamber.

The model working electrode (WE) comprises a physically vapor-deposited thin film of Ni, subsequently annealed to form an electron-conducting 2D network with a spectroscopically accessible and electrochemically active TPB of the metallic film, the YSZ substrate, and the gas phase. The detailed preparation of this cell concept is described in a previous work.^20^ This study focuses on the comparison of two specifically designed patterned model electrode structures. “Structure A” was prepared by the aforementioned single thermal annealing step.^20^ In contrast, “Structure B” was the result of a subsequent redox cycling procedure (+1.0 to −0.4 V) at 550 °C in a 1 mbar H_2_:H_2_O = 1:9 gas atmosphere carried out in the NAP-XPS chamber, which led to progressive dewetting by oxidizing and reducing the Ni thin film, thereby forming Structure B. In terms of the electrochemical stability of the Ni thin film, CV cycling under predominantly cathodic conditions (+0.1 to −1.5 V; 1 mbar H_2_:H_2_O = 1:9) between 400 and 550 °C was not accompanied by measurable changes of the Ni 2p intensity, indicating that much less surface compositional/structural changes are induced if the Ni thin film remains metallic (Figure S1). Figure 1A exemplifies a scanning electron microscopy (SEM) image combined with an energy-dispersive X-ray spectroscopy (EDXS) map (Ni K) of the Ni/8-YSZ WE surface, illustrating the percolated Ni^0^ film (∼100 nm thick) and the YSZ islands of Structure A.

The cell setup was assembled using a Pt mesh (CE) between thermally insulating quartz glass rings on a stainless-steel NAP-XPS sample holder (Figure 1B). A K-type Ni/NiCr thermocouple was thermally contacted to the YSZ substrate by H_2_-sintering the wires together with a Ni paste droplet next to the Ni thin-film electrode. This allows for control of the sample temperature via the heating power of a pulsed diode IR laser irradiating the CE side of the cell.

### Characterization Methods

#### WE Characterization

Two distinctly structured WE thin films were characterized by scanning electron microscopy (SEM). The topographic and EDXS imaging of Structure A shown in Figure 1A was performed on an FEI Quanta 650 FEG SEM (acceleration voltage of 15 kV, high vacuum conditions at around 5 × 10^–6^ mbar). Structure B (Figure S2) was characterized by topographic and EDXS imaging using a ZEISS Ultra 55plus FEG SEM at an electron acceleration voltage of 20 kV. Structure A exhibits a predominantly closed Ni thin film with YSZ pit-holes, while Structure B features a ramified Ni domain network among much larger YSZ surface domains. Additional topological imaging was obtained by atomic force microscopy (AFM, Dimension 3100, Veeco), employing n-doped silicon probes in tapping mode (1.2 V amplitude, force constant of 20–80 N m^–1^, resonance frequency of 256–317 kHz, RTESPW, Veeco). The AFM images cover an 80 μm × 80 μm section and were recorded with a resolution of 1024 × 512 data points and a scan rate of 0.15 Hz. Structural and geometrical data (i.e. TPB length, YSZ surface coverage, and YSZ domain size) were extracted from the SEM and AFM images by using the software package Gwyddion 2.64.^27^

**Figure 2:**
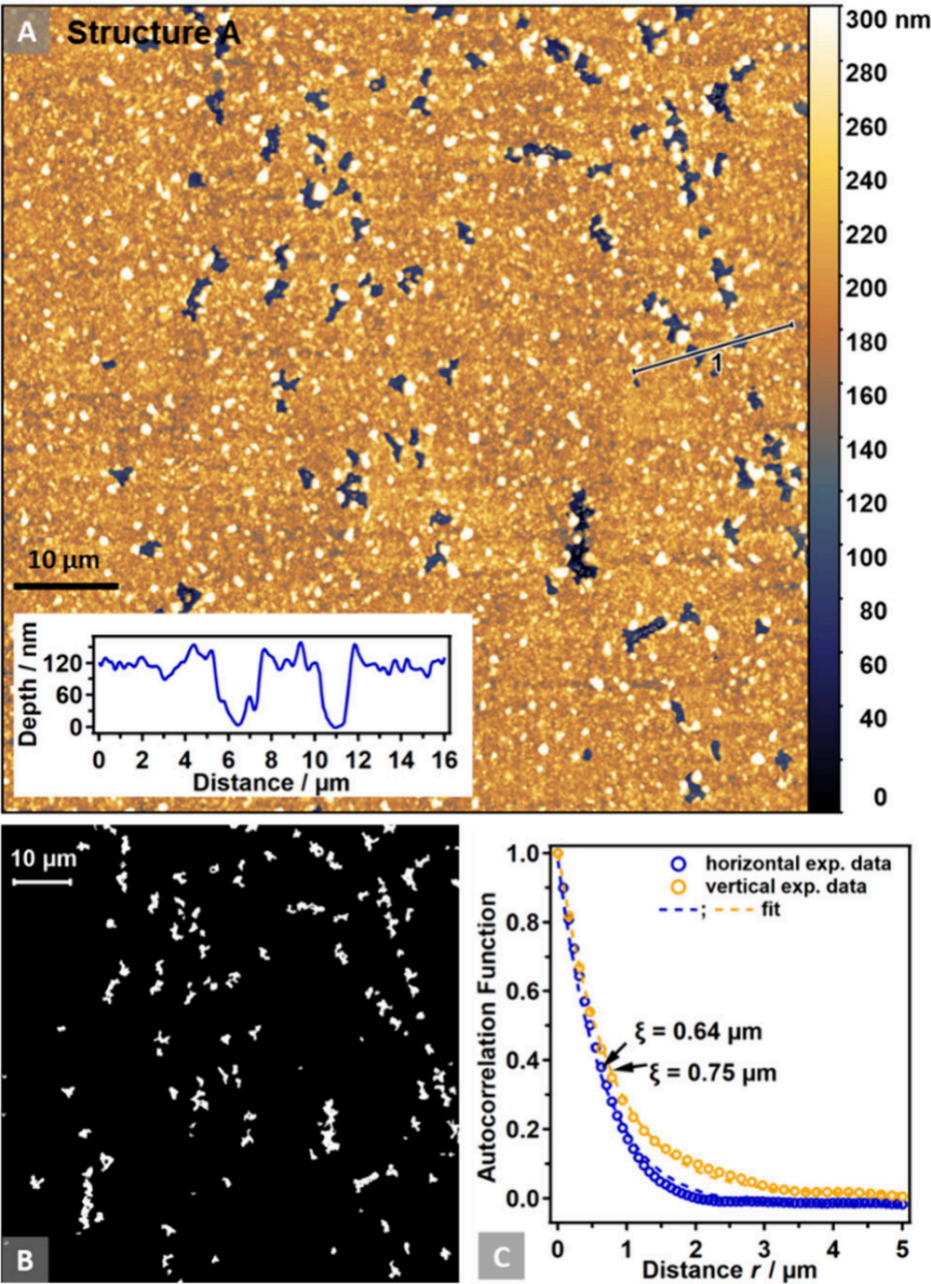
(A) AFM image (80 μm × 80 μm) depicting the 2D porous Ni thin film of Structure A on 8-YSZ. The inset shows a height profile crossing two pit-holes within the nominally 100 nm thick Ni film. (B) Black (Ni) and white (YSZ) domain patterns of the image in (A). (C) Autocorrelation fitting of the domain pattern in horizontal and vertical directions.

The TPB length and YSZ surface area were determined by masking the respective YSZ area in the top-view images and subsequently extracting the area and circumference of the masked regions^20^ (Figure S2). The YSZ domain size was calculated according to a study from Dürrbeck et al.,^28^ where, as a prerequisite, the masked image is converted into a black (Ni) and white (YSZ) domain pattern. The 1D autocorrelation function (ACF) of the pattern can be computed in the horizontal and vertical directions, and its progression yields an exponential decay over the distance *r*.^28,29^
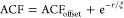
2A fit of the computed ACF yields the correlation length ξ, which is a measure of the YSZ domain size.^28^ The procedure is visualized in Figure 2. For error calculations of the YSZ domain size and the TPB length, refer to the Supporting Information (Note S1).

#### *Operando* NAP-XPS, MS, and EIS Setup

For electron spectroscopic *in situ* investigations under HER reaction conditions, experiments were conducted in a laboratory-based SPECS NAP-XPS system.^30^ The ultrahigh vacuum (UHV) chamber comprises a μFOCUS 600 NAP monochromatic small spot (300 μm) Al–K_α_ X-ray source, a hemispherical energy analyzer (SPECS PHOBIOS 150 NAP equipped with a 1D delay line detector) in a vertical configuration, and a μ-metal main chamber shielding the system from external electric and magnetic fields. The differentially pumped electrostatic lens system separates gas molecules from the photoelectrons focused toward the hemispherical energy analyzer, enabling XPS experiments during backfilling of the analysis chamber to pressures up to 25 mbar with variable gases and gas mixtures via mass flow controllers (Bronkhorst). Two quadrupole mass spectrometers (QMS, Hiden HPR-40 and MKS Instruments e-vision2) are installed in the first and second differential pumping stages past the analyzer nozzle, respectively, and operated depending on the specific sensitivity requirements to measure the gas phase composition and products formed during the *in situ* XPS experiments. Gases evolving at the sample surface are pumped through the nozzle and can be detected online without delay, providing live information on product formation at the sample surface. An IR laser (max 120 W) is attached to the bottom side of the analyzing chamber, allowing precise heating of the samples from the backside via an 8 mm hole in the sample holder. The X-ray source power was set to 70 W and 13 kV, while the analyzer was set to a constant pass energy of 50 eV for all recorded spectra. A potentiostat-equipped electrochemical impedance (EIS) spectrometer (Biologic, SP200) connected to the WE and CE contacts of the sample holder was utilized to control the electrochemical conditions and to investigate the dynamics of charge-transfer and non-charge-transfer processes. EIS spectra were obtained in the frequency range from 1 MHz to 20 mHz, while the perturbation amplitude was set to 5 mV. The EIS data were analyzed using the Zahner Z-Hit Analysis software package. All XPS data were analyzed using the CasaXPS software, version 2.3.24 PR1.0 (Casa Software Ltd.). For peak fitting, a Shirley background was applied to all spectra. Fitting of the O 1s species was done using a weighted sum of Gaussian and Lorentzian peak shapes with a GL(30) contribution. The Ni 2p_3/2_ peak can be associated with a binding energy (BE) of 852.6 eV for the metallic component,^30^ while the BE of the Zr 3d_5/2_ peak is close to 182.8 eV, indicating the Zr^4+^ state in an 8-YSZ environment.^30^ The characteristic O 1s region of YSZ consists of two components.^31^ The lower BE peak (∼530 eV) is associated with lattice oxide (O–Zr(Y)), while the higher BE peak (∼532 eV) is attributed to hydroxide oxygen species.^32^ For the determination of the YSZ surface coverage from the XPS data, refer to the Supporting Information (Note S2).

#### Computational Methods

The software package COMSOL Multiphysics 6.1 was utilized to simulate the local electric field (e-field) within the electrolyte during polarization in the vicinity of the TPB. The model was constructed by reconfiguring an excerpt of the cross section of the cell. A 150 μm thick YSZ electrolyte with a relative permittivity of 26.4^33^ and a CE with a thickness of 10 μm were incorporated. The WE structure features were based on the results obtained from SEM and AFM analyses. Specifically, the measured extensions of the YSZ domains were utilized to simulate the average lateral YSZ distance between the 100 nm thick Ni domains (Figure S3). To approximate the potential drop across the electrolyte, the CE was set to +1.0 V, while the WE was grounded. The model employed a resolution of 1 mV for the equipotential lines. A triangular grid was utilized, wherein the e-field strength was determined at each grid point. To ensure a manageable computation time, a flexible triangular size starting at 10 nm and increasing at a rate of 1.3 to a maximum of 1 μm was employed.

## Results and Discussion

### Structural Parameters of the WE

The geometrical and structural properties of the thin Ni film layer and the YSZ substrate can be categorized as follows (for their determination, refer to the WE Characterization section):

(a) TPB length: the geometrical length of the TPB, which represents the interface between the Ni thin-film layer, the YSZ substrate, and the gas phase.
(b) YSZ surface coverage: the total Ni-free YSZ surface fraction available for interaction with the gas phase.
(c) YSZ domain size: average diameter of Ni-free YSZ domains, particularly significant due to its effect on the local electric field, derived from a correlation-length analysis of the available AFM and SEM data.

Figure 2 illustrates the analysis based on these categories for the model electrode with Structure A, based on the respective AFM image. Figure 2A displays the topological AFM image, showcasing the percolated Ni thin film after the annealing procedure described in ref (20), as well as nominally 100 nm deep pit-holes uncovering the YSZ substrate (cf. Figure 1A). This pore depth is confirmed by the inset, which represents a profile through such a feature. By masking the pit-holes, the TPB length and YSZ surface coverage were determined.^20^ After the mask is converted into a black (Ni) and white (YSZ) domain pattern (Figure 2B), a 1D autocorrelation function can be computed in both horizontal and vertical directions from the pattern (Figure 2C). Applying eq 2 to fit the data yields the correlation length ξ, which is associated with the most frequently occurring distance where a domain change occurs. ξ is therefore a measure of the YSZ domain size.^28,29^

**Figure 3:**
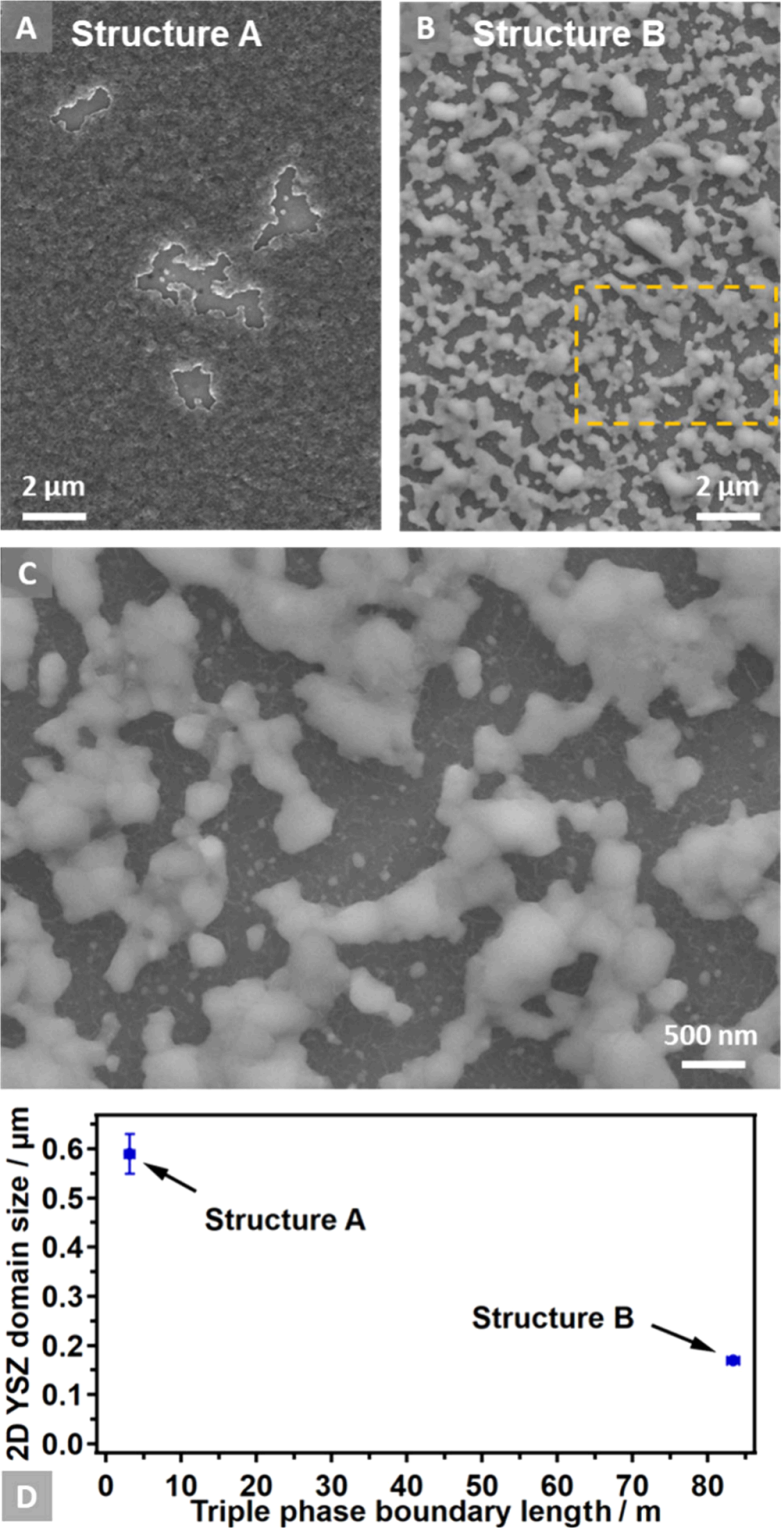
(A–C) SEM images of the Ni/YSZ thin-film model electrode, represented as Structures A and B, captured at different magnifications. (D) Averaged results from the SEM and AFM image analyses illustrating the averaged YSZ domain size and TPB length of the two investigated structures.

The intention behind the design of thin Ni film structures on YSZ was to create variations in structural parameters. This made a detailed microscopic investigation and analysis necessary, as exemplified in Figure 3. A comparison of the top-view SEM images of the metal thin films of Structure A (Figure 3A) and Structure B (Figure 3B) allows for a computational assessment of the differences in TPB length, YSZ surface coverage, and YSZ domain size of these two model electrodes. Notably, in Structure B, the Ni domains appear more dispersed due to stronger dewetting. Therefore, Figure 3C provides a magnified view of a region of Structure B, revealing still highly percolated Ni domains with a minor presence of isolated Ni particles. However, similar isolated Ni features within the thin-film cavities were observed in Structure A, too.

Figure 3D presents the averaged results of a series of AFM and SEM image analyses. The YSZ domain sizes of Structure A (0.59 ± 0.04 μm) and Structure B (0.17 ± 0.01 μm) are plotted against the TPB lengths of Structure A (3.1 ± 0.6 m) and Structure B (83.3 ± 0.8 m).

The determination of the YSZ surface coverage completes the investigation of the structural parameters. Table 1 compares the YSZ surface coverages of Structures A and B as determined by XPS and SEM/AFM analyses. Both structures yielded consistent results in both analytical methods. The small discrepancy in the analysis of Structure B is likely due to the variations within the limited probe area (SEM: ∼1800 μm^2^, AFM: ∼6400 μm^2^ vs XPS: ∼30 000 μm^2^) of each technique, which falls within the range of <0.15% of the total button cell area, thus rendering local deviations likely. However, we regard the results to be reliable enough to promote further interpretations.

**Table 1:** YSZ Surface Coverage of Structures A and B Determined by XPS and SEM/AFM Analyses

	XPS analysis	SEM/AFM analysis
sample	YSZ surface coverage (%)	
Structure A	3 <± 1	3 ± 1
Structure B	42 <± 1	39 ± 1

### Structure- and Polarization-Dependent Surface Hydroxylation under HER Conditions

A series of NAP-XPS experiments conducted in a H_2_:H_2_O 1:9 mixture (1 mbar) at isothermal conditions of 400, 450, and 500 °C unveiled a voltage-induced surface response. During the experiments, XPS spectra of the Ni 2p, Zr 3d, and O 1s regions were recorded while the cell was held at open-circuit voltage (OCV) or at a constant applied voltage (*E*_WE_ = −1.0 V) over time. Especially under cathodic conditions, where water reduction and the HER are active, NAP-XPS measurements enable the monitoring of the electrochemically induced surface composition and redox state.

**Figure 4:**
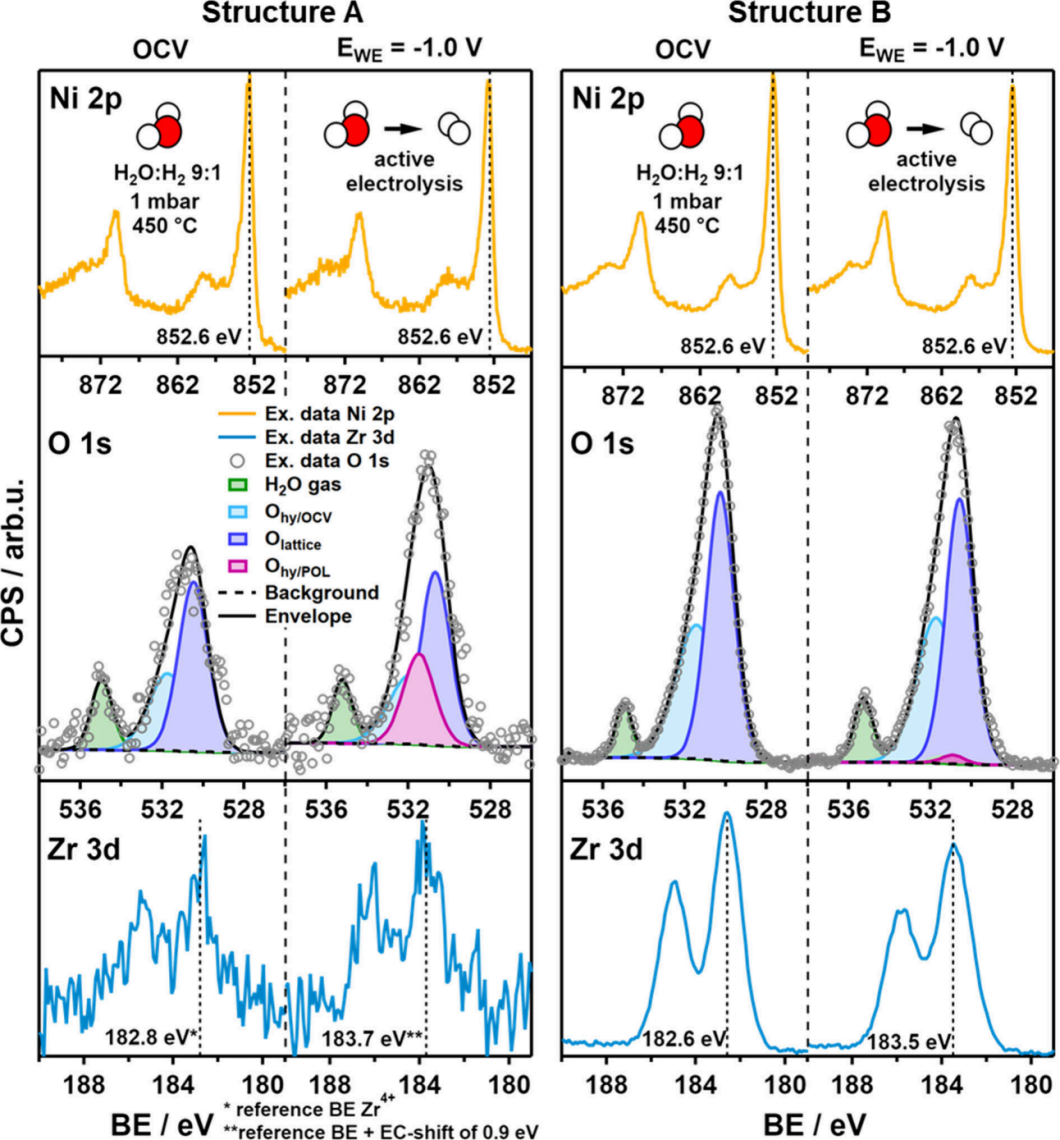
XPS data of the Ni 2p, O 1s, and Zr 3d regions, recorded for Structures A and B of the Ni/8-YSZ thin-film model electrodes and compared with respect to the polarization of the electrode (OCV vs *E*_WE_ = −1.00 V). The plots represent the data detected at 450 °C in a 1:9 H_2_:H_2_O mixture (1 mbar). In the O 1s region at around 532 eV, an additional feature, O_hy/POL_, was added to account for the main intensity response to cathodic polarization.

Figure 4 compares the response of each electrode’s surface state (i.e., of Structures A and B) upon cathodic polarization, as revealed by conducting simultaneous NAP-XPS measurements under isothermal conditions of 450 °C.

While no changes upon polarization were detected in the Ni 2p region, as the conductive Ni film is grounded alongside the spectrometer, the Zr 3d peak appears at a ∼0.9 eV higher binding energy, indicating the expected shift of the electronically insulating YSZ due to the applied polarization.^30^ Due to the low YSZ surface coverage fraction of Structure A, the signal-to-noise ratio of the Zr 3d and O 1s regions is limited. However, the O 1s peak of Structure A undergoes drastic changes upon setting the voltage to *E*_WE_ = −1.0 V, whereas only minor changes were recorded for Structure B.

For quantifying this effect, the O_lattice_ (530.4 ± 0.2 eV) and O_hy/OCV_ (531.6 ± 0.2 eV, Zr–OH without polarization) components, as described in ref (32), were consistently constrained in full width at half-maximum (fwhm) and BE across all XPS spectra taken under OCV conditions (Table S1). Based on the polarization-induced shift of the H_2_O(g) component, the O_lattice_ and O_hy/OCV_ peaks were allowed to shift accordingly in order to fit the experimental data detected under cathodic conditions.^34^ By conserving the integral peak areas of these peaks, an additional component associated with cathodic polarization-induced surface hydroxide (O_hy/POL_) was added to the fit. Since the YSZ-related O 1s intensity rises drastically relative to the gas phase water O 1s intensity, which only depends on the constant water partial pressure, and the shape of the O 1s peak changes markedly at the high BE side around 532 eV, the addition of this component appears highly justified. As cathodic polarization induces additional oxygen surface vacancies, the reaction of water molecules at these sites most likely results in this additional O_hy/POL_ species. Alternative explanations of this enhanced O 1s intensity are hardly conceivable when considering the enhanced oxygen vacancy-mediated surface chemistry of YSZ in the presence of water vapor upon cathodic polarization.

In a control experiment at 550 °C with a “reproduced” Structure A-type electrode, polarization-dependent surface hydroxylation was again observed (Figure S4). By progressively polarizing the WE cathodically at the OCV, *E*_WE_ = −0.5 V, and *E*_WE_ = −1.0 V, a simultaneous growth of the hydroxide component in the O 1s region was detected, confirming a potential-dependent surface hydroxylation response.

The same procedure was carried out for all model electrode structures and temperatures, and the results are summarized in Table 2. The increase of the O_hy/POL_ contribution by turning the cathodic polarization on and off was found to be highly consistent within the respective electrode motif (Structure A or B) and largely independent of temperature (Table 2). Based on these results, we interpret the large differences in the polarization-induced YSZ surface hydroxylation as a result of the different model electrode structures. For details regarding the error analysis of the XPS fitting routine, refer to the Supporting Information (Note S3).

**Table 2:** Summary of the NAP-XPS Data Analysis of the O 1s Regions of Structures A and B at the Given Temperatures under Cathodic Polarization, along with the Average Atomic % of O_hy/POL_ for Each Model Electrode Structure

sample	*T* (°C)	O_hy/POL_ (at. %)	O_hy/OCV_ (at. %)	O_lattice_ (at. %)
Structure A	400	27.5 ± 2.5	26.9 ± 1.1	45.7 ± 1.8
	450	25.9 ± 2.3	27.5 ± 1.1	46.7 ± 1.9
	500	25.6 ± 2.3	27.6 ± 1.1	46.9 ± 1.9
average ± standard deviation		26.3 ± 1.4		
Structure B	400	1.8 <± 0.1	36.4 <± 0.4	61.7 <± 0.6
	450	1.4 <± 0.1	43.0 <± 0.4	55.6 <± 0.6
	500	2.3 <± 0.1	36.0 <± 0.4	61.7 <± 0.6
average ± standard deviation		1.8 ± 0.2		

### Dependence of HER Activity on Electrode Structure

From the hydroxylated YSZ area detected during the NAP-XPS experiments, which is interpreted to be equal to the water-activating fraction of the total available (i.e., Ni-free) electrolyte area, and the structural knowledge of the purely geometrical TPB length, as determined from the SEM/AFM data analysis, the lateral extension of the near-TPB “water-activating zone”, namely, the TPB width, was calculated for each electrode structure (Note S4). The argumentation of an increased activity close to the TPB is supported by the findings of Horita et al., which demonstrate that the polarization-induced exchange of O^18^ isotopes is limited to the vicinal area of the TPB.^35^Table 3 contrasts the structural data and sets the hydroxylated TPB area in relation to the total (Ni-free) YSZ area. In addition, ratios of the structural parameters, either A/B or B/A, are provided. The bold values will be useful for the interpretation of the EIS data shown later.

**Table 3:** Comparison of TPB Parameters with Respect to Structures A and B

sample	YSZ_area_ (mm^2^)	TPB_area_ (mm^2^)	TPB_length_ (m)	TPB_width_ (m)
Structure A	0.64 ± 0.13	0.17 ± 0.03	3.1 ± 0.6	52.6 ± 5.5
Structure B	7.71 ± 0.13	0.14 ± 0.01	83.3 ± 0.8	1.7 ± 0.0
ratio A/B	0.08	1.2	0.04	31
ratio B/A	**12**	**0.8**	**27**	0.03

**Figure 5:**
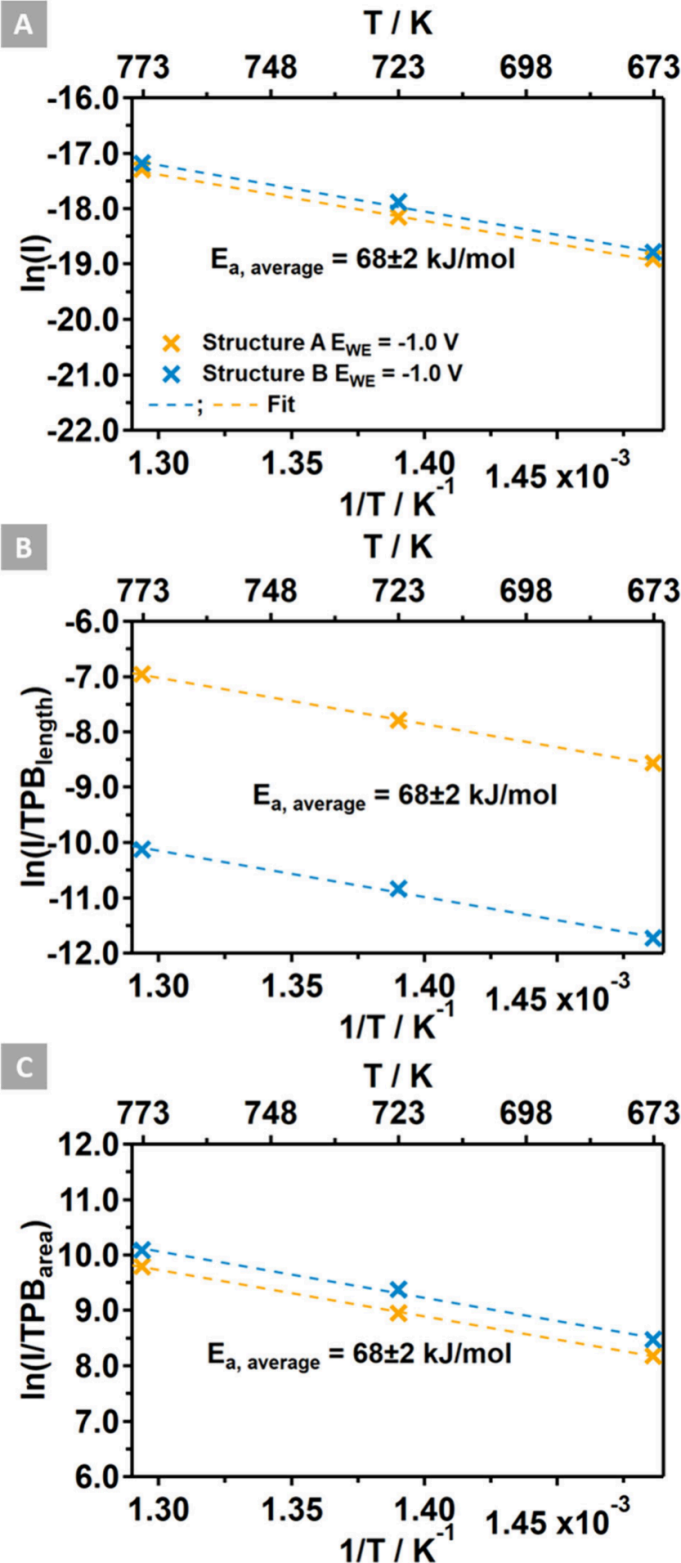
(A) Arrhenius plot for the unnormalized current. (B) Arrhenius plot for the current normalized to the geometrical TPB length. (C) Arrhenius plot for the current density at the hydroxylated surface area. The activation energy annotated in the plot was averaged for both model electrodes (Structures A and B).

On this basis, the electrode activities measured during cathodic polarization for each structural motif and temperature were normalized to either the TPB length or the TPB area, resulting in the Arrhenius plots shown in Figure 5. The simplest concept of an active TPB anticipates that the current should be a function of the geometrical TPB length (i.e., a single row of YSZ/Ni sites) and is usually normalized to this structural parameter. Surprisingly, the unnormalized total currents on Structures A and B were quite similar (Figure 5A). Normalization by the TPB length resulted in current densities that differ by almost 2 orders of magnitude (Figure 5B). If the hydroxylated YSZ area derived from the NAP-XPS experiments is taken as a measure for a near-TPB “water catching” area, assuming fast inverse spillover of H atoms to Ni and H_2_ desorption from adjacent metallic sites, then the current density anomaly can be resolved by normalizing the total current to this structural parameter to yield a local current density. The Arrhenius plot depicted in Figure 5C elucidates the results of this approach and suggests an effective zone of the TPB wider than a quasi-linear YSZ/Ni site row. This TPB area encompasses the entirety of electrochemical activity and has its origin in the structural motif (i.e., Structure A vs Structure B) of the model electrode. The activation energy obtained by fitting the experimental data was very similar on both structures (A and B) and averaged to 68 ± 2 kJ/mol. This suggests the occurrence of a common local water reduction mechanism, characterized by identical sites and transition states, which appears to be independent of the structural motif of the electrode. However, the attempt frequency (i.e., the pre-exponential) is contingent upon the real number of available water activation sites. The structural parameter controlling this number is, therefore, the near-TPB electrolyte surface area rather than the TPB length.

The chosen approach aims to illustrate that the HER activity does not adhere strictly to the concept of a structure–activity relation scaling with the TPB length alone. Despite considering a “water catching” area near the TPB, interpretations of the kinetics or even reaction mechanisms based solely on a one-step reaction from H_2_O to H_2_ (eq 1) are quite unrealistic. Rather, a sequence of individual elementary reactions prevails, as discussed in the literature.^16,17^ In this context, the EIS characterization will reveal a more complex situation by considering different elementary steps and their time scales within the overall electrochemical reaction (eq 1).

### E-Field Modeling

**Figure 6:**
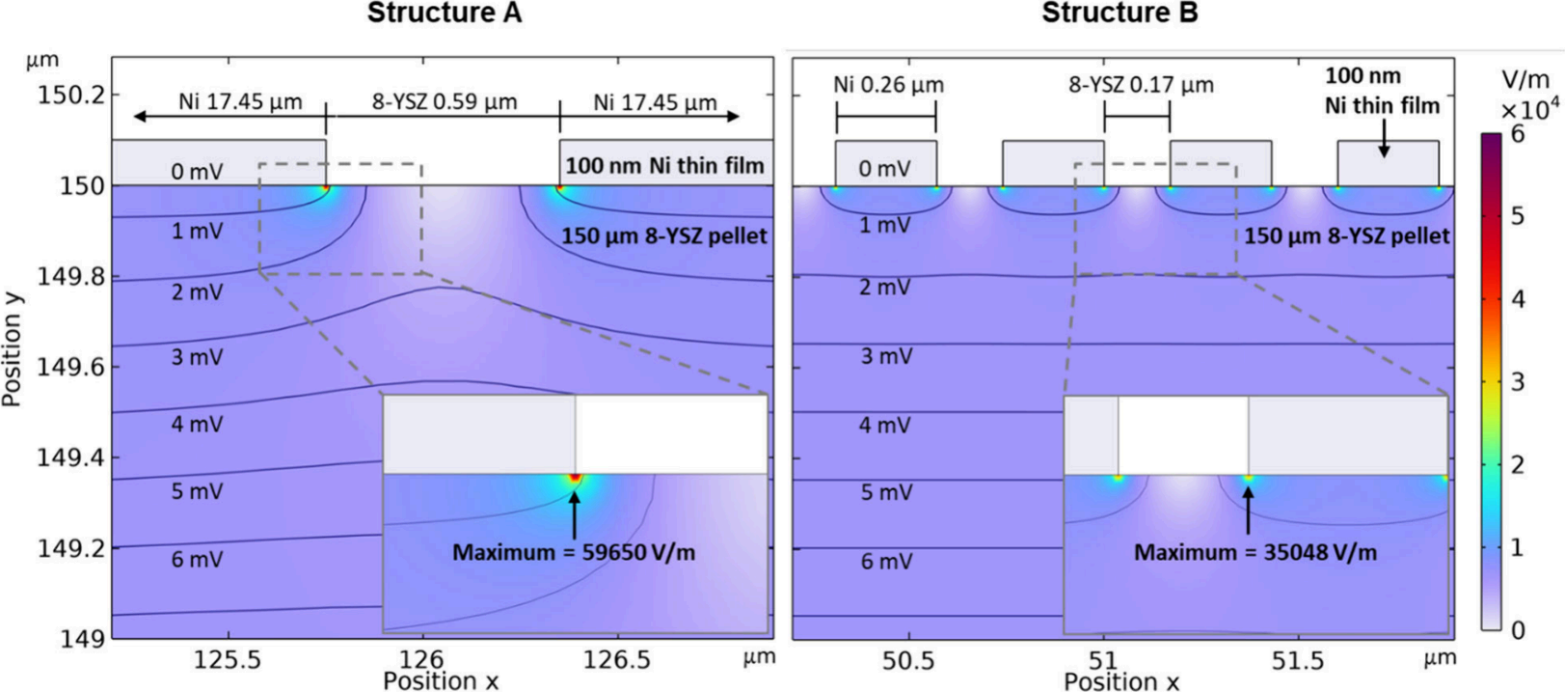
A magnification of the cell’s cross section depicting the TPB region at the WE. The local e-field is determined by the simplified electrode pore Structures A (left) and B (right). The e-field strength is presented according to a color code legend. The inset magnifies the respective TPB zone and indicates the maximum strength of the e-field. The equipotential lines are generated from the simulated potential drop between the CE and WE of 1 V.

The question of how the TPB structure can influence electrochemical activity was addressed through an e-field modeling approach computed by means of the COMSOL Multiphysics 6.1 software package. For this, a cross section through the cell, including the CE, 150 μm YSZ electrolyte, and 100 nm WE thin film, was constructed (Figure S3). The domain sizes of the Ni layer and YSZ were obtained from structural characterization (cf. Figure 3) and are annotated in Figure 6. The crucial structural parameter controlling the e-field strength was identified as the YSZ domain size. Given that the Ni layer becomes negatively charged under cathodic polarization, the migration of hydroxy and oxygen ions (OH^–^ and O^2–^) away from the Ni/YSZ interface is likely. However, proton-hopping based on surface hydroxylation is well-documented for YSZ up to 523 °C.^36−39^ Consequently, potential-induced surface hydroxylation represents an ideal prerequisite for such a mass transport mechanism. The activation energy for surface H^+^ migration is dependent on the H_2_O partial pressure, leading to literature values ranging from 24 to 50 kJ/mol,^37,40^ which is considerably lower than ∼105–115 kJ/mol for O^2–^ ion transport through the electrolyte.^37,41^ The literature suggests a predominant contribution of proton conductivity up to 400 °C^38^ and even up to 500 °C under humidified conditions.^37^ Based on literature findings and the degree of hydroxylation on the TPB-vicinal YSZ surface, verified by NAP-XPS, we can assume that the strength and progression of the e-field play a major role in controlling the electrochemical activity, facilitating an effective charge-transfer reaction (CTR) according to eq 3 in the close vicinity of the TPB. Additionally, they contribute to the accumulation of oxygen vacancies in this area, thereby facilitating effective surface hydroxylation. This behavior was observed on patterned model Pt/YSZ electrodes by Janek et al. using scanning photoelectron microscopy.^14^
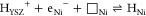
3In this perspective, eq 3 is facilitated on Structure A compared to Structure B due to the difference in the local e-field strength (Figure 6).

In the following, an estimate of the differences of the near-TPB electric potential is provided. The movement of H^+^ along the surface from a position *x* toward the TPB edge is associated with a potential energy change, which can be determined by using eq 4:
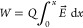
4Simulations in the literature^16^ suggest that higher surface coverages of hydroxyls appear close to the TPB (<10 nm), which aligns more closely with the situation in Structure B, with a TPB width of *x* = 1.7 nm. Based on this, the energy *W*_B_ according to eq 4 can be calculated. Upon examining the position in the *x*-direction in Structure A, which exhibits the same e-field strength cutoff as in Structure B, we find *x* = 8.7 nm and an energy *W*_A_. The ratios of these energies (*W*_A_/*W*_B_ = 8 and ***W***_**B**_**/*W***_**A**_**= 0.13**) are crucial structure-based values utilized in the interpretation of the EIS data. Due to the predominance of proton conductivity, the differences in energy between *W*_A_ and *W*_B_ are regarded as a measure controlling the CTR (eq 3).

### Electrochemical Impedance Spectroscopy

**Figure 7:**
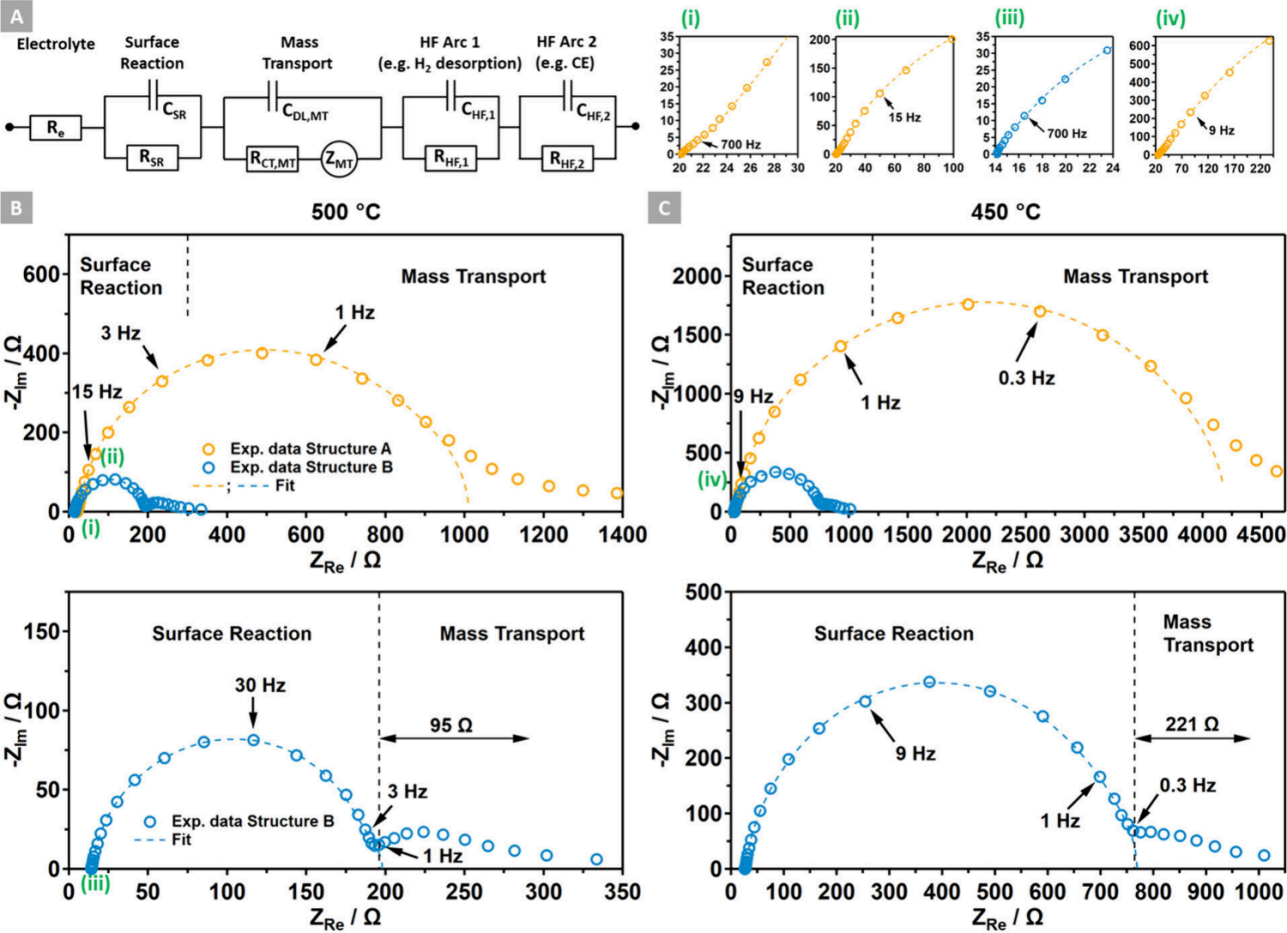
(A) Equivalent circuit for fitting the EIS data and resolving the frequency-dependent reaction steps. (B, C) The EIS experimental data and fit of Structure A (orange) are compared to the respective dataset of Structure B (blue). The EIS data were recorded at (B) 500 °C and (C) 450 °C in a 0.1 mbar H_2_:H_2_O 1:9 mixture. The magnifications on the top right illustrate the fit quality in the higher-frequency range as indicated by the Roman numerals (i) to (iv).

To encompass all relevant elementary reaction steps, an equivalent circuit model for patterned model electrodes according to ref (42) was adapted, which accurately fits the EIS data. The circuit, formalized in Figure 7A, identifies a series of four RC elements representing the following mechanistically necessary elementary processes:

(i) YSZ surface hydroxylation, followed by a charge-transfer (CT) process with a negligible mass transport limitation (short TPB width case: Structure B).
(ii) Mass transport-controlled CTR (high TPB width case: Structure A).
Both processes generate H_ads_ species according to eq 3 on the adjacent Ni surface, which undergo rapid associative H_2_ desorption. The last two RC elements are associated with high-frequency processes estimated as the following:
(iii) Associative H_2_ desorption from the Ni surface.
(iv) Relatively fast exchange reaction on the CE.

The serial electrolyte and wiring resistances (*R*_e_) are modeled as a single resistor, but one needs to consider that the electrolyte contribution is a function of temperature and the contribution of the wiring is constant. Due to the construction of the NAP-XPS manipulator, this constant contribution of ∼17 Ω is relatively large and exceeds the electrolyte resistance at 500 °C (*R*_e_ = 17 + 3 = 20 Ω; values for Structure A; Table S4). At 400 °C, the total serial resistance *R*_e_ amounts to 17 + 30 = 47 Ω. A change of the electrolyte resistance by a factor of ∼10 is expected for this temperature range.^41^ However, the approach of a serial circuit of RC elements allows one to assign a certain frequency to each elementary reaction step,^43−45^ justifying each RC element physically. The mass transport (*Z*_MT_)-controlled CTR (*R*_CT,MT_, *C*_DL,MT_) appears at a very low frequency (<1 Hz, 500 °C, OCV) and can be separated from a surface reaction-driven CT process (*R*_SR_, *C*_SR_) occurring in the mid-frequency range (5–30 Hz, 500 °C, OCV). The contributions from the associative desorption of H_2_ (*R*_HF,1_, *C*_HF,1_) and the CE (*R*_HF,2_, *C*_HF,2_) are found in the area of high to very high frequencies (100 Hz–1 kHz, 500 °C, OCV). These frequency ranges are consistent with literature values found for similar temperatures on patterned model electrodes.^17,42^

According to the literature,^16,44,46,47^ the impedance data recorded at OCV conditions and presented in Figure 7B,C reveal a diffusion-controlled CTR for the electrode Structure A (orange trace). In this scenario, the comparatively low TPB length is the structural parameter controlling the mass transport-related CT resistance *R*_CT,MT_,^48^ subsequently limiting the current. In this context, it is proposed that the mass transport process involves the migration of ionic species (e.g. H^+^) in the electric field,^16,37,39^ rather than purely thermal diffusion of suspected H_ads_ species on YSZ. Another notion is also discussed in literature, which suggests the presence of hydrogen on YSZ in its molecular form H_2_.^36^ For Structure B (blue trace), which exhibits a 27 times higher TPB length and a 31 times shorter TPB width, the diffusion limitation largely disappears, and processes associated with a direct gas/surface reaction-controlled CT are steering the current flow.^16,17,39,46^ Consistently, in a surface reaction-controlled mechanism, the H^+^ diffusion length decreases significantly (cf. Table 3), staying close to the TPB.

The frequency/time constant resolved for every elementary step was found to be consistent for both model electrode structures. Nonetheless, the EIS data established on a symmetrical cell (Pt/GDC//YSZ//Pt/GDC), which primarily demonstrates a diffusion-controlled electrochemical reaction (Figure S5), were utilized to correct the mass transport resistance determined on the Ni/YSZ thin-film cells. Contributions from the CE in the frequency range of the CTR were negligibly small (500 °C: 140 Hz, ∼2 Ω and 400 °C: 15 Hz, ∼9 Ω) and therefore not considered for further corrections, in consistency with ref (24). Figure 7B,C compares the EIS data for Structures A and B recorded at 500 and 450 °C, confirming a very low frequency contribution for the diffusion-controlled pathway for Structure A and a mid-frequency contribution of the surface reaction pathway on Structure B. For the 400 °C data, refer to Figure S6. The fit of Structure A includes the full equivalent circuit from Figure 7A. However, data points obtained below 100 mHz were excluded from the fit due to difficulties in adapting the fit to the progression of the respective data points in this frequency range. These characteristics are well-established in corrosion research, where inhibitors that block active sites lead to progressively increased resistance while decreasing the frequency.^49^ Since the EIS experiment was conducted under OCV conditions, the temporary modulation of the voltage toward the anodic regime becomes notably significant in the lower-frequency spectrum. Given that the redox chemistry of Ni manifests itself near the OCV^20^ rather than under strongly cathodic conditions and assuming a partially irreversible Ni oxidation, progressive Ni site inhibition near the TPB appears plausible and may contribute to the observed phenomenon in the low-frequency range of the mass transport segment. This oxidation could potentially serve as an inhibitor, contributing to the observed phenomenon. The EIS data of Structure B distinguish the residual mass transport part from the surface reaction part. Therefore, due to the aforementioned fitting issue, the equivalent circuit was adapted, and the mass transport elements were excluded from the fitting procedure. Instead, the mass transport resistance (*R*_CT,MT_ + *Z*_MT_) of Structure A was divided by the ratio of the TPB length between B and A (ratio_B/A_ = 27, cf. Table 3) resulting in the WE contribution of Structure B to mass transport effects. By summing up this value with the mass transport resistivities of the CE, an estimate for the complete mass transport contribution of Structure B was obtained (Table S2). Figure 7 illustrates this estimate in the mass transport part of the EIS data for Structure B, matching the experimental data quite well.

Table S2 presents the results of the EIS data fitting procedure for Structures A and B at each temperature. The table associates a frequency with each (electro)chemical elementary process, which is very similar on both Structures A and B. Beneath each process frequency, the EIS fitting model proposes an elementary process resistance *R* and capacitance *C* for each structure. As the EIS characterization was conducted under OCV conditions and, therefore, at negligible overpotentials, the low field approximation of the Butler–Volmer equation can be formally utilized to relate the current density to the reciprocal charge-transfer resistance *R*_CT_:^19,47^
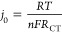
5

The plot of ln (*I*_CT_) ∝ ln(*T/R*_CT_) versus 1/*T* shows Arrhenius behavior and allows to extract an activation energy. This procedure, conducted in the literature for mass transport- and surface reaction-controlled CT reactions,^19,47^ enables one to determine the thermal activation of electrochemical polarization processes (e.g., surface reaction including CT) and electrolyte kinetics.^39^ Accordingly, we consider *R*_SR_ and *R*_CT,MT_ as resistive elements following this approximation (eq 5) and plot the calculated current densities as a function of temperature.

**Figure 8:**
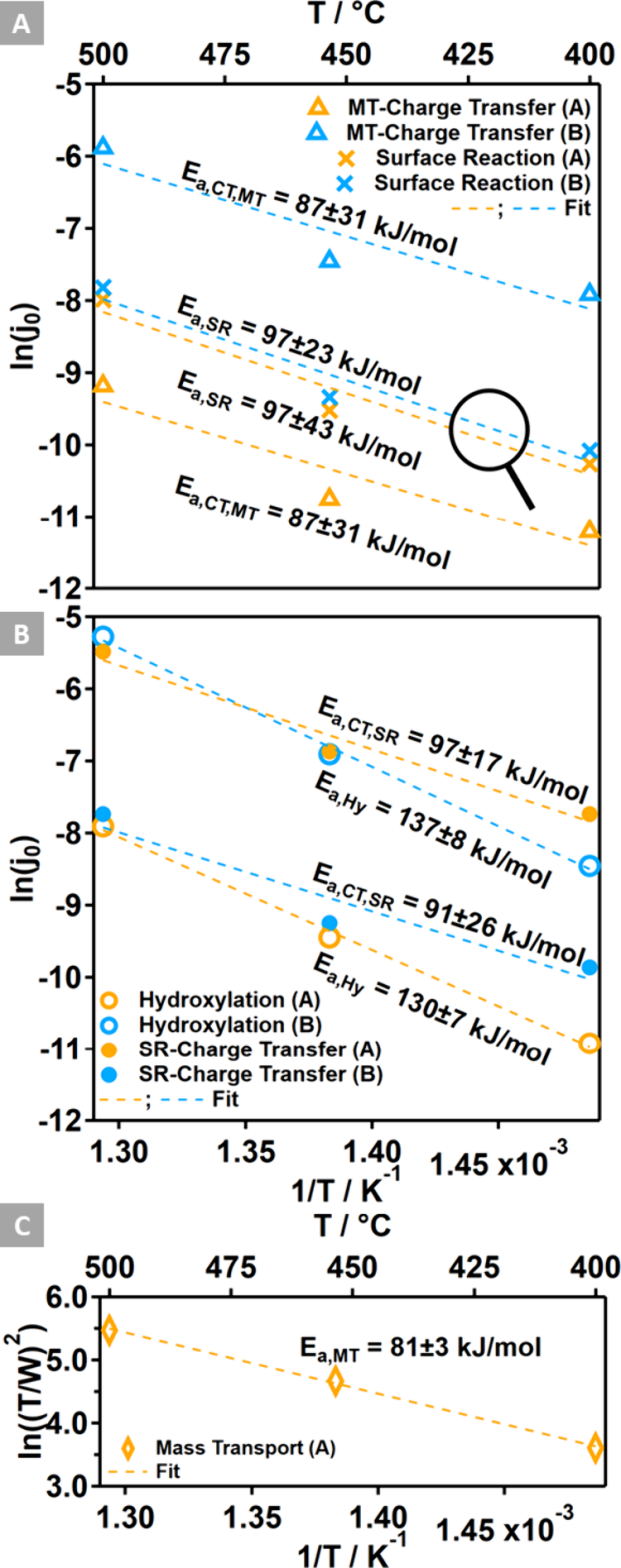
Arrhenius plots derived from the EIS data fits, which resolve the following elementary processes and associate an activation energy with them: (A) the mass transport-controlled CTR and surface reaction including the CT process. (B) The surface reaction further resolved into a surface hydroxylation step and a CTR controlled by the hydroxylation. (C) The thermal activation of the mass transport process.

Figure 8A illustrates the results of this analysis, indicating the lowest activity for the mass transport-controlled CTR on Structure A, followed by a similar activity of the surface reaction-controlled CT process on both Structures A and B, completed by the CT reaction controlled by diffusion on Structure B. Thus, the picture derived from the EIS plots in Figure 7 can be confirmed, reflecting the backlog of surface hydroxyl species in Structure A compared to a near-TPB hydroxylation as detected by NAP-XPS.

The activation energy of 87 kJ/mol for the mass transport-controlled CTR is not very much beyond the reported literature values of 72 kJ/mol^47^ and 77 kJ/mol.^45^ However, it has been shown that the partial pressures of H_2_O and H_2_ significantly influence the activation energy, with values of 110 kJ/mol^19^ to 132 kJ/mol^42^ being reported as well. A thermal activation of 97 kJ/mol was determined for the surface reaction including the CT, which is closer to the data from ref (39) predicting 85 kJ/mol.

The surface reaction-controlled CTR limits the current in Structure B, necessitating a separation of these two elementary processes. This was achieved by establishing two RC elements representing the YSZ surface hydroxylation and charge-transfer reaction. This deconvolution improves the rather rough frequency range of 4–30 Hz (500 °C) for the *R*_SR_/*C*_SR_ element toward a distinct lower frequency of 3 Hz for the hydroxylation element (*R*_Hy_/*C*_Hy_) and a clearly higher frequency of 15–30 Hz for the charge-transfer element (*R*_CT,SR_/*C*_DL,SR_), being consistent on Structures A and B (Table S3).

The resistances and capacitances of the RC elements related to associative H_2_ desorption and CE exchange reactions as well as the ohmic resistance associated with electrolyte and wiring are summarized in Table S4.

Figure 8B illustrates the thermal activation of resistors *R*_Hy_ and *R*_CT,SR_, representing the surface hydroxylation and subsequent CTR, respectively. As Structure B exhibits a comparatively high YSZ surface area and a pronounced TPB length, the process limiting the current is the CTR. In contrast, the current in Structure A is limited first by mass transport and second by surface hydroxylation, consistent with the limited TPB length and YSZ surface area, respectively. A comparative analysis of the EIS parameters (*R*_Hy_ and *R*_CT,SR_) between Structures A and B identifies similarities with the structural parameters of the WE, relating the structure with the critical elementary reaction step.

For the YSZ surface hydroxylation reaction occurring at a frequency of 1 Hz (400 °C), 1.5 Hz (450 °C) or 3 Hz (500 °C) on Structures A or B, the resistance drops by a factor of 12 to 14 (A → B), consistent with the increase in YSZ surface coverage by a factor of 12 (A → B). Anticipating a similar reaction probability, being predominantly a function of the H_2_O(g) impingement rate, we speculate that each water molecule encounters the 12-fold area for adsorption in Structure B compared to that in Structure A, leading to a proportional drop in resistance.

A similar behavior was observed for the CT process, which appears to correlate with the electric potential present in the vicinal area of the TPB region (cf. E-Field Modeling section). The resistance of the CTR at the TPB of Structure A is 11 to 8 times smaller as compared to that of Structure B, consistent with the differences in the energy of the near-TPB electric field, which is 8 times higher in Structure A compared to Structure B. This suggests that a structure-based change, namely the extension of the YSZ domain size, affecting the near-TPB e-field strength, would potentially result in a reduction of the CT resistance. This reduction could lead to an improvement in activity by more than 2 orders of magnitude, as illustrated in Figure 8B.

The derived activation energies of 91 and 97 kJ/mol for the surface reaction-controlled CT process are very similar to the value determined for the mass transport-controlled CTR of 87 kJ/mol. Literature values for a surface reaction-driven CT range from 70 up to 150 kJ/mol.^19^

For the potential-induced YSZ surface hydroxylation, a thermal activation of 130 to 137 kJ/mol was determined, which agrees reasonably with literature values citing ∼120 kJ/mol.^17^

Additionally, the fit of a Nernst element (*Z*_MT_) in the mass transfer part yielded the possibility to determine an activation energy for the mass diffusion process.^48^ The impedance of this element is defined as
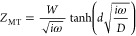
6Besides the frequency ω, the finite diffusion length *d*, and the diffusion coefficient *D*, the *W* in eq 6 represents the Warburg impedance, which is defined as
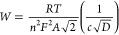
7In eq 7, *c* is the concentration of the diffusing species, and *A* is the surface area of the model electrode. Rearrangement yields
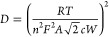
8Assuming a constant concentration of the diffusing species, as detected by NAP-XPS (cf. Table 2) and condensing all other constants yields
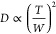
9Therefore, a method to extract the thermal activation of the diffusion process can be provided, as shown in Figure 8C. The fitted overall Warburg impedance was corrected by the Warburg impedance of the CE derived from the EIS data of the symmetrical cell (Table S2 and Figure S5), yielding the Warburg impedance of the WE, which was used for the Arrhenius plot. The determined activation energy of 81 kJ/mol ranges between that of H^+^ diffusion with an activation energy of 24 to 50 kJ/mol^37,40^ and that of O^2–^ bulk diffusion with a thermal activation of 105 to 115 kJ/mol.^37,41^ We speculate that a local charge separation process, involving the field-driven attraction of H^+^ along the increasingly negative field toward the TPB and, vice versa, the lateral repulsion of O^2–^ ions in the opposite direction, contributes to this activation energy.

We emphasize that a clear correlating trend between frequencies and temperatures can be extracted from the numerical values of the fit parameters summarized in Tables S2 and S3. These trends can only be reproduced in a physically meaningful way if the full set of physically justified RC elements is used for the fit procedure, thus confirming the chosen approach with up to six serial circuits for fitting the EIS data of our patterned model electrodes. In addition, the correlation of the resistance and capacitance of each individual reaction process to a given structural parameter provides further support for the chosen set of physically justified RC elements.

**Figure 9:**
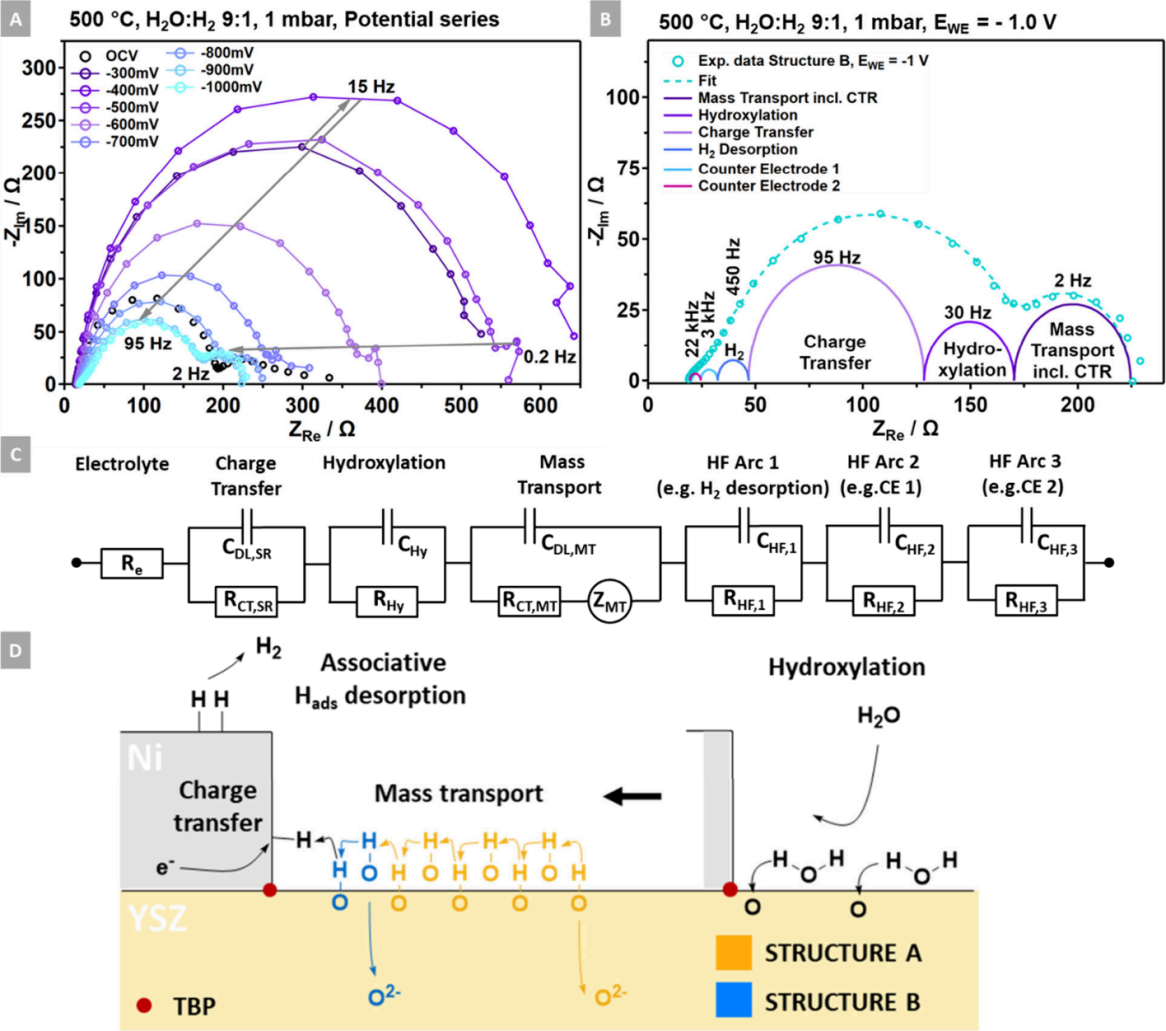
(A) EIS data recorded during a stepwise polarization series from OCV to −1000 mV. The experiment was conducted on Structure B at 500 °C in a H_2_O:H_2_ 9:1 mixture of 1 mbar. (B) EIS data and fit of the spectrum recorded at −1000 mV. The different elementary processes described by five RC elements and one (R-Z)C element (Z = Nernst diffusion impedance) are indicated. (C) Equivalent circuit used for fitting the EIS data. (D) Proposed mechanism showcasing two distinct reaction pathways. One pathway, depicted in orange, emphasizes a significant contribution from mass transfer tailored to Structure A. The other pathway, shown in blue, features a negligible diffusion branch, reflecting the conditions observed on Structure B.

In relation to the literature,^39,42^Figure 9 illustrates the changes of the impedance as a function of cathodic polarization at 500 °C on Structure B. Figure 9A shows a significant increase of the polarization resistance between OCV and −400 mV, followed by a substantial decrease in resistance while increasing the polarization to −1000 mV. Starting with a rather noisy signal in the mass transport region at 0.2 Hz, this feature evolves into a well-shaped arc with a frequency of 2 Hz due to the stepwise increase in polarization. Furthermore, the “tailing” assigned to site inhibition by partial Ni oxidation, observed in the mass transport segment under OCV conditions (Figure 7), disappears already at voltages around −300 mV, thus supporting the argumentation of time-dependent anodic Ni deactivation in the low-frequency range. The main arc exhibits a frequency of 15 Hz, identifying this feature as the surface hydroxylation-controlled CTR (Table S3). As the cathodic potential increases, the frequency shifts toward 95 Hz, consistent with the literature.^39,42^

Figure 9B represents the experimental data and fit of the spectrum recorded at −1000 mV. The combination of high-frequency processes, a hydroxylation-controlled CT, and a mass transport-limited CTR yields a proper fit of the experiment data, resolving the rate-limiting step as a charge-transfer reaction, according to eq 2. In Figure 9C, the equivalent circuit utilized to fit the experimental data is depicted. It comprises the previously mentioned RC elements for hydroxylation (*R*_Hy_/*C*_Hy_) and the surface reaction-driven charge-transfer process (*R*_CT,SR_/*C*_DL,SR_). Additionally, the circuit incorporates a third high-frequency arc (*R*_HF,3_/*C*_HF,3_; 22 kHz), whose contribution to the overall is minor but which is inevitable to properly fit the high-frequency shoulder. Nonetheless, including this arc aligns well with the experimental data and may stem from an unknown CE process activated solely under anodic polarization. The data derived from the fit are summarized in Table S5.

From the combination of the experimental observations and the subsequent data analysis, a reaction mechanism for the hydrogen evolution reaction (HER) in electrolysis mode is proposed in Figure 9D. The elementary reaction steps, particularly the H^+^ migration, align with a study by Bieberle et al. focusing on the hydrogen oxidation reaction (HOR) in fuel cell mode.^39^ The mechanism depicts YSZ surface hydroxylation by H_2_O(g) as the initial reaction step, followed by mass transport. The degree of hydroxylation and the efficiency of H^+^ hopping are correlated with the YSZ surface area, the TPB geometry, and the near-TPB e-field strength, aligning with the two distinct pathways for Structures A (orange) and B (blue), as validated by NAP-XPS. Subsequent charge-transfer processes (cf. eq 3) produce H_ads_ species on the adjacent Ni surface, which then undergo rapid associative desorption to form H_2_(g).

## Conclusions

This study underscores the importance of the electrode structure, as its local structural properties influence the near-TPB electric field, leading to significant consequences for the current flow. A structure prone to diffusion-controlled charge-transfer processes with limited TPB length and YSZ surface area exhibits the same activity (i.e., cell current) as a structure with superior TPB length and YSZ surface area but a weaker near-TPB e-field strength. This weakness manifests itself in the surface hydroxylation-controlled charge-transfer reaction, which limits the current. An idealized 2D model electrode structure would consist of an extended YSZ domain size (>0.5 μm) while maintaining superior TPB length and YSZ surface area properties (cf. Figure 3D). Such a structure would potentially improve the cell current by 2 orders of magnitude. The interplay between the electrode structure and its activity manifests itself in several ways: (i) the YSZ domain size-dependent e-field controlling the charge-transfer reaction in close vicinity to the TPB, (ii) the supply of mass toward the TPB, which is limited by the TPB length, and (iii) the YSZ surface area influencing the surface hydroxylation pathway.

In this context, the transfer of mechanistic knowledge based on patterned model electrodes to the optimization of the performance of applied porous cermet electrodes is difficult to estimate, as additional structural parameters such as grain size, pore size, and percolation of the metallic and oxidic domains must be considered.

## Supporting Information Available

Ni thin-film stability, structural analysis of the working electrode from microscopic characterization, error calculation of the YSZ domain size and TPB length, analysis of the YSZ surface coverage from XPS characterization, approach to model the near-TPB e-field properties, constraints in the NAP-XPS data analysis of the O 1s region, error analysis of the XPS fitting procedure, determination of the TPB width, EIS analysis of the symmetrical cell (Pt/GDC//YSZ//Pt/GDC) and the thin-film cells at 400 °C, and EIS analysis results (PDF)
