# Supplementary material for: New Insights into the Hydrogen Evolution Mechanism near the Ni/YSZ Triple Phase Boundary during Steam Electrolysis: A Patterned Model Electrode Study
Source: ACS Electrochem. 2024 Nov 4;1(3):315–27. doi: 10.1021/acselectrochem.4c00031 (PMC11891889; doi:10.1021/acselectrochem.4c00031)
Supplement: Supplementary file 1 — ec4c00031_si_001.pdf [file ec4c00031_si_001.pdf]

# Supporting Information

## New insights into the hydrogen evolution mechanism near the Ni/YSZ triple phase boundary during steam electrolysis: A patterned model electrode study


Christoph W. Thurner[1,3,*], Kevin Ploner[2], Daniel Werner[1], Simon Penner[1], Engelbert Portenkirchner[1] and Bernhard Klötzer [1,*]

[1]*Institute of Physical Chemistry, University of Innsbruck, Innrain 52c, A-6020 Innsbruck, Austria*

[2]*Plansee SE, Metallwerk-Plansee-Strasse 71, 6600 Reutte, Austria*

[3]*Ceratizit S.A, 101 Rue de Holzem, 8232 Mamer, Luxembourg*

[*] Corresponding Authors: [E-mail: christoph.thurner@ceratizit.com; bernhard.kloetzer@uibk.ac.at]


**Table of Contents**


Ni thin-film stability upon cathodic cycling in 1 mbar $H_2$:$H_2O$ = 1:9 (non-redox with respect to Ni) ................S2
Structural analysis of the working electrode from the microscopic characterization ..........................................S2
Error calculation of the YSZ domain size and TPB length ..........................................S3
Analysis of the YSZ surface coverage from the XPS characterization ..........................................S4
Approach to model the TPB-near e-field properties ..........................................S5
Constraints in the NAP-XPS data analysis of the O 1s region ..........................................S6
Potential-dependent surface response detected by NAP-XPS ..........................................S6
Error analysis of the XPS fitting procedure ..........................................S7
Determination of the TPB-width ..........................................S8
EIS analysis of the symmetrical cell ((Pt/GDC///YSZ//Pt/GDC)) ..........................................S9
EIS analysis of the thin-film cells at 400 °C ..........................................S10
Results of the EIS analysis ..........................................S11
References ..........................................S14

**Ni thin-film stability upon cathodic cycling in 1 mbar $H_2$:$H_2O$ = 1:9 (non-redox with respect to Ni)**

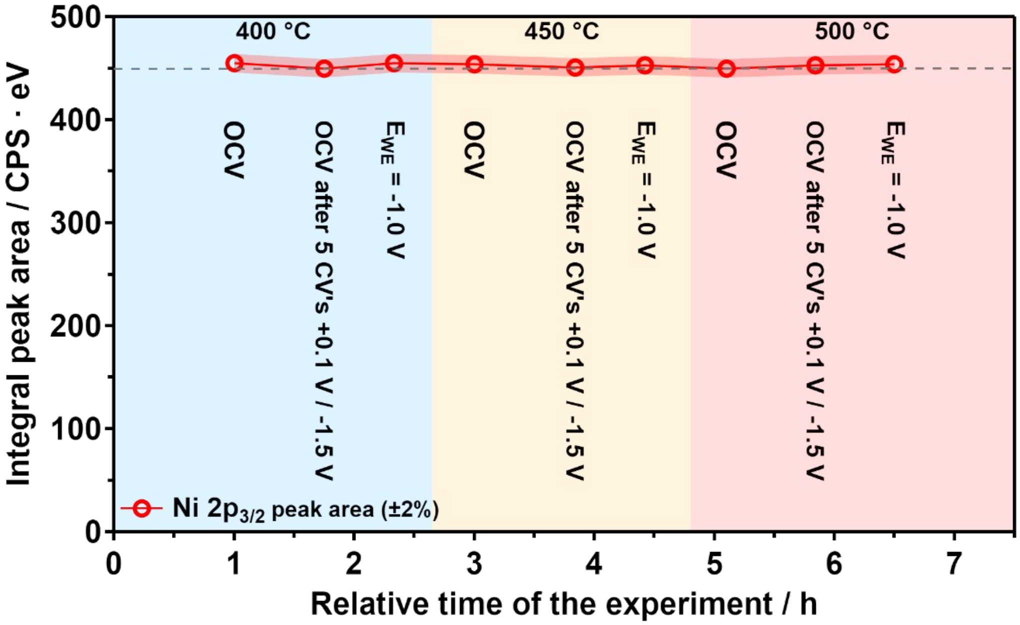


**Figure S1.** For the series of NAP XPS experiments carried out on Structure A, the integral peak area of the Ni $2p_{3/2}$ region (red circles) is plotted as a function of time and potential. The color shaded-areas represent the chosen temperature of the WE, while polarization and pretreatment of the electrode are indexed below each data point.

**Structural analysis of the working electrode from the microscopic characterization**

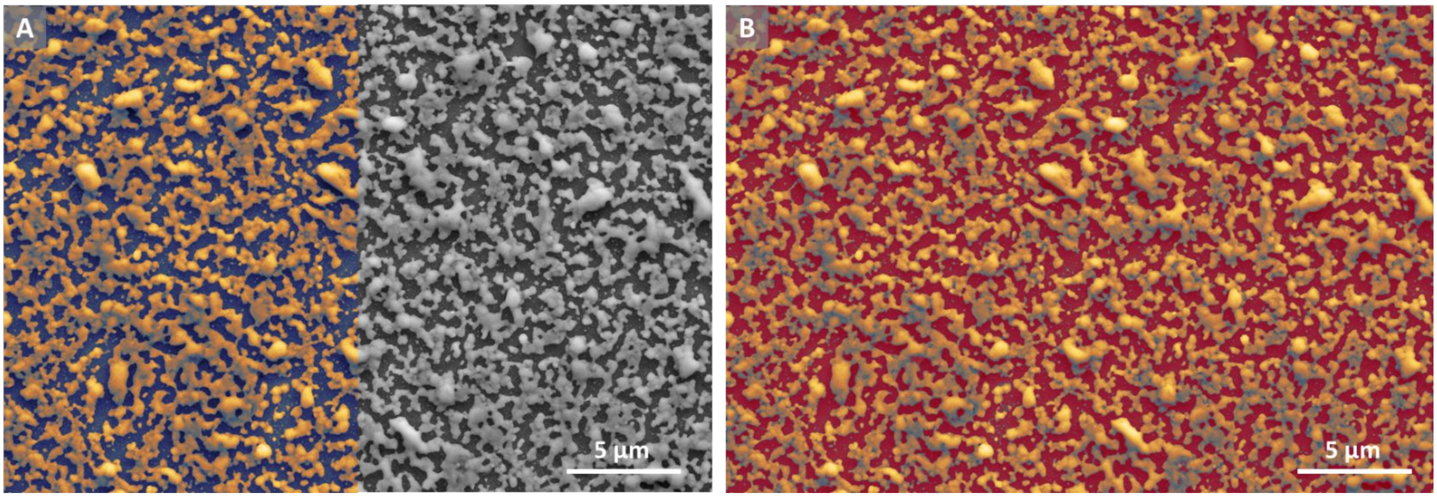


**Figure S2.** A: SEM image of Structure B displaying the percolated Ni domains (highlighted in yellow or light grey) and the YSZ substrate (displayed in blue or dark gray). The colors have been adjusted from the original grayish image. B: Using the mask covering the YSZ surface, the software program (Gwyddion 2.64 [1]) calculates

the circumference, which corresponds to the TPB length, and the area, which represents the YSZ surface area.

## Error calculation of the YSZ domain size and TPB length

**Note S1:**

The respective error values have been calculated based on a series of AFM and SEM image analyses. The pit holes are exhibit all a very similar depth because of the uniformly thick starting film grown on a flat, polished YSZ substrate. Tests at different positions showed a uniform step height at the pit walls. The size of the individual pit holes is very similar as well. However, as the further geometrical analysis is focused on the lateral dimensions, we applied an error calculation to these parameters. The variation of the lateral parameters, especially the YSZ domain size and the TPB length was taken into account as follows:

(1) YSZ domain size (correlation-length): The YSZ domain size was determined by averaging the correlation-length derived from 8 individual 1D auto correlation function fits. The error of the average $u_{\bar{\xi}}$ was determined according to:

$$u_{\bar{\xi}} = \sqrt{\frac{\sum (\xi - \bar{\xi})^2}{n \cdot (n-1)}} \qquad (1)$$

$\xi$ is the correlation-length of the individual 1D auto correlation function fit. $\bar{\xi}$ is the average of the individual correlation-lengths determined. $n$ is the amount of individual data points used for the calculation of the average.

(2) TBP length: The TBP length was determined by summing up the circumference of the each pit hole in the AFM or SEM image. The result form each image analysis was then averaged. The error of the average $u_{\overline{TPB}}$ was determined according to:

$$u_{\overline{TPB}} = \sqrt{\frac{\sum (TPB - \overline{TPB})^2}{n \cdot (n-1)}} \qquad (2)$$

$TPB$ is the TPB-length of the individual image analysis. $\overline{TPB}$ is the average of the individual TPB-lengths determined.

## Analysis of the YSZ surface coverage from the XPS characterization

**Note S2:**

According to [2], the determination of the YSZ surface coverage from the XPS data proceeded as follows: The integral peak area of the Ni $2p_{3/2}$ and Zr 3d signal was divided by the atomic density of the respective XPS-active layer ($\rho_{i,layer}$), yielding the surface contribution of Ni and Zr domains and, therefore, the YSZ coverage. The atomic density of the XPS-active layer of the respective specimen was calculated using the formula:

$$\rho_{i,layer} = \rho_i \cdot \Lambda_i(E_{kin,i}) \qquad (3)$$

Thereby, $i$ denotes the specimen (Ni or Zr) and $\rho_i$ represents the atomic density in atoms/$cm^3$. It is noteworthy that for Zr, the density of Zr atoms in 8-YSZ was calculated. The variable $\Lambda_i(E_{kin,i})$ stands for the effective attenuation length (EAL) of the Ni 2p or Zr 3d photo-electron with the respective kinetic energy. The EAL was derived from the SRD 82 data base [3]. For Ni and Zr, an atomic density of the XPS-active layer of $6.92 \times 10^{15}$ atoms/$cm^2$ and $8.17 \times 10^{15}$ atoms/$cm^2$ was determined. The YSZ surface coverage was calculated from each individual XPS data set for each temperature (400 °C, 450 °C and 500 °C) under both OCV and cathodic conditions. The standard deviation of the average was negligible and accounted for an error in the YSZ surface coverage of less than 0.01.

## Approach to model the TPB-near e-field properties

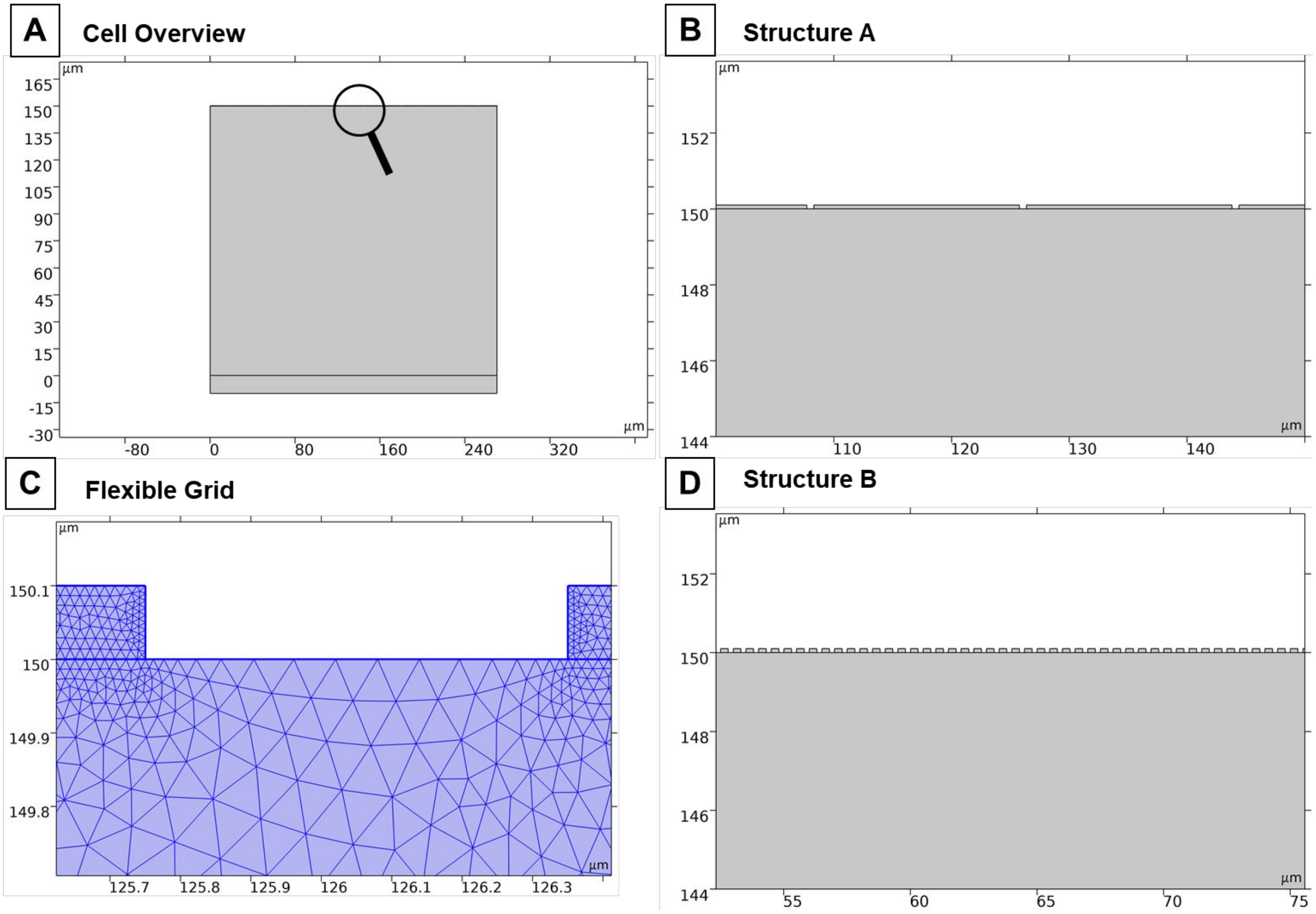


**Figure S3.** Panel A: An excerpt of the cell's cross section was modelled, including a 150 µm thick electrolyte layer (center), a 10 µm thick counter electrode layer (bottom) and a 100 nm thick working electrode (top). Panels B and D depict magnifications of the WE, illustrating the domain pattern of Structure A and B. Panel C shows the development of the flexible grid between two Ni domains.

### Constraints in the NAP-XPS data analysis of the O 1s region

**Table S1.** Constraints of the full-width-half-maximum (FWHM) and the binding energy (BE) position of the O1s components fitted under OCV conditions and cathodic polarization.

| Component | BE OCV /eV | FWHM / eV | BE POL /eV |
|---|---|---|---|
| $O_{lattice}$ | 530.4 ± 0.1 | 1.0 - 1.7 | + 0.3 |
| $O_{hy/OCV}$ | 531.6 ± 0.1 | 2.2 - 2.5 | + 0.3 |
| $H_2O$ gas | 535.0 ± 0.1 | 0.9 - 1.2 | + 0.3 |
| $O_{hy/POL}$ | - | 1.3 - 1.8 | 531.5 ± 0.1 |

### Potential-dependent surface response detected by NAP-XPS

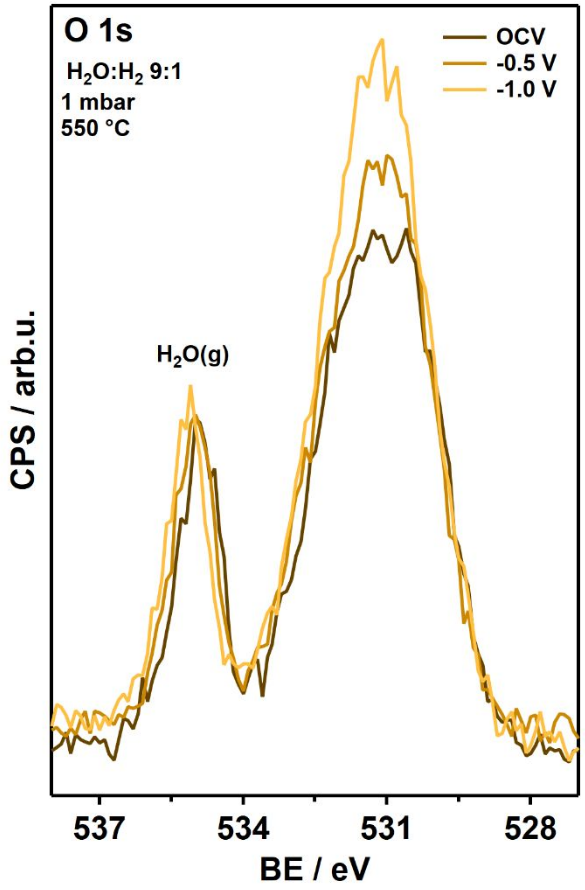


**Figure S4.** XP spectra of the O 1s regions recorded on a structural motif similar to Structure A of the Ni/8-YSZ thin-film electrode. The surface response to the step-wise cathodic polarization (OCV, $E_{WE}$ = -0.50 V and $E_{WE}$ = -1.00 V) is evident in the increase of the hydroxide component. The data was detected at 550 °C in a $H_2$:$H_2O$ 1:9 mixture (1 mbar).

## Error analysis of the XPS fitting procedure

**Note S3:**

The error in the determination of the peak area within a spectrum exhibiting a rather noisy background, results from the choice of the integration boundaries, which influence the position of the background function in y-direction. Errors originating from the choice of the background function itself can be seen as a systematic error. By using consistently the same background function for the analysis of each spectrum, such error contributions did not lead to any significant failure when comparing systematically error-prone spectra. Consequently, throughout this study a Shirley background was applied. However, a variation of the integration boundaries in the quantitative analysis of the O 1s, Zr 3d and Ni 2p peak of structure A (Figure 2) showed the following results:

The variation of the peak area by varying the integration boundaries is ≈± 4% in the case of the O 1s peak, ≈± 9% for the Zr 3d peak and ≈± 2% for the Ni 2p peak. In this context, the uncertainty of the O 1s peak area of ≈±4% is only valid for the O 1s main component at ≈533 eV. The uncertainty of the $H_2O$(g) component is comparable to the Zr 3d uncertainty of ≈±9%, which further effects the uncertainty of the O 1s peak area increase upon polarization. To account for this fact the $O_{hy/POL}$ component, which is associated to the polarization-induced increase of the O 1s peak area is estimated with an uncertainty of ≈±9% as well.

Consequently, the YSZ surface coverage of structure A presented in Table 1 exhibit an error of ≈± 9% and the O 1s components $O_{hy/OCV}$ and $O_{lattice}$ of structure A presented in Table 2 are error-prone by ≈± 4%, while the O 1s component $O_{hy/POL}$ is associated with an uncertainty of ≈± 9%. The variation of the peak areas of the O 1s , Zr 3d and Ni 2p regions of structure B were considerably lower at <± 1%.

The standard deviation of the average of the individual $O_{hy/POL}$ components (Table 2) is determined by:

$$u_{\overline{O_{hy/POL}}} = \sqrt{\frac{\sum (O_{hy/POL} - \overline{O_{hy/POL}})^2}{n \cdot (n-1)}} \qquad (4)$$

$O_{hy/POL}$ is the peak area of the respective component in the O 1s region. $\overline{O_{hy/POL}}$ is the average of the individual peak areas. $u_{\overline{O_{hy/POL}}}$ is the standard deviation of the average. $n$ is the amount of individual data points

used for the calculation of the average. However, if one considers the propagation of uncertainty of independent variables the error is calculates by:

$$u_{O_{hy/POL}} = \frac{\sqrt{u_{O_{hy/POL,1}}{}^2 + u_{O_{hy/POL,2}}{}^2 + u_{O_{hy/POL,3}}{}^2}}{n} \quad (5)$$

$u_{O_{hy/POL,i}}$ is the error of the peak area of the respective component in the O 1s region. $u_{O_{hy/POL}}$ is the error from the propagation of uncertain individual data points (i.e., the average calculation). In this specific case, the errors of equation (4) with ±0.6 at% is lower than error calculated by equation (5), which accounts for ±1.4 at%, as given in Table 2.

## Determination of the TPB-width

**Note S4:**

The NAP-XPS data analysis presented in Table2 show the electronically-induced hydroxylation of the YSZ-surface, which is associated with the detected $O_{hy/POL}$ component upon cathodic polarization. The variable $\overline{O_{hy/POL}}$ is the average of the $O_{hy/POL}$ components detected at three temperatures (Table 2) and represents the proportion of the total YSZ surface ($YSZ_{area}$ in Table 3) which is active upon polarization. Therefore, the active YSZ surface fraction ($TPB_{area}$) is calculated by:

$$TPB_{area} = YSZ_{area} \cdot \frac{\overline{O_{hy/POL}}}{100} \quad (6)$$

The $TPB_{area}$ is divided by the $TPB_{lenght}$ to yield the $TPB_{width}$:

$$TPB_{width} = \frac{TPB_{area}}{TPB_{lenght}} \quad (7)$$

The TPB parameters are presented in Table 3.

## EIS analysis of the symmetrical cell ((Pt/GDC///YSZ//Pt/GDC))

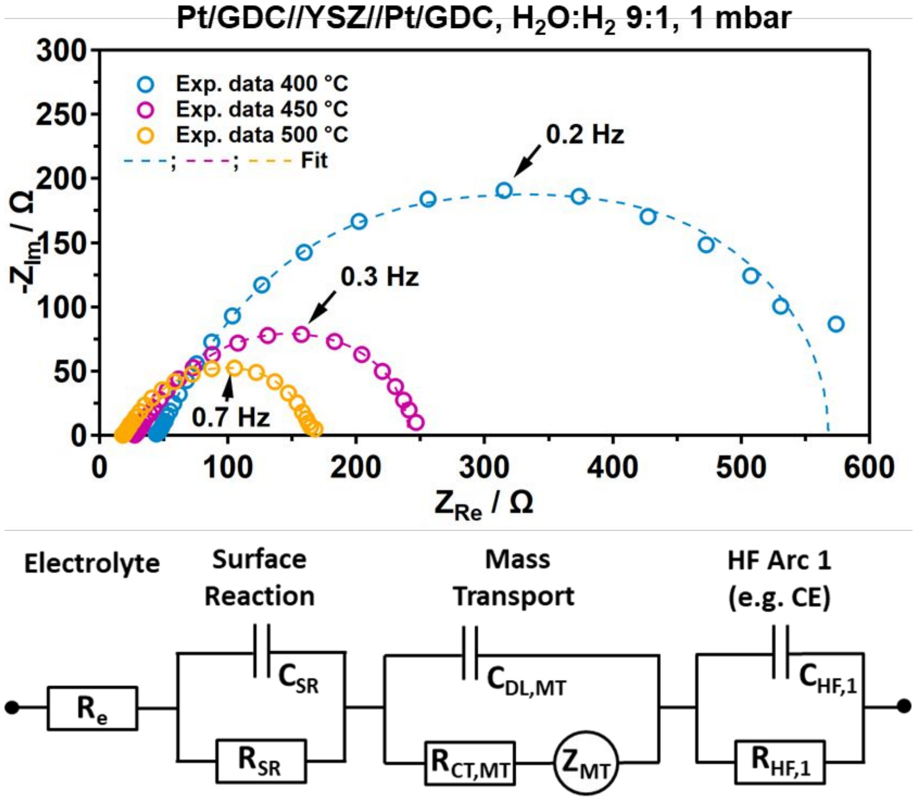


**Figure S5.** The EIS data were recorded under the same conditions and on the same instrument (NAP-XPS) as used for investigating the thin-film cells. The manufacturing process and the raw materials of the cell remained consistent with those of the thin-film cells. However, instead of the thin-film WE electrode, a WE consisting of a porous Pt/GDC-10 layer covered by a pure Pt current collector layer was prepared. Due to the relatively low frequency of the main arc, a mass transport-controlled charge transfer reaction is expected. The parameters, particularly the resistances, extracted by fitting the experimental data with the depicted equivalent circuit, were halved and applied to correct the mass transport data obtained from the EIS characterization of the thin-film cells.

## EIS analysis of the thin-film cells at 400 °C

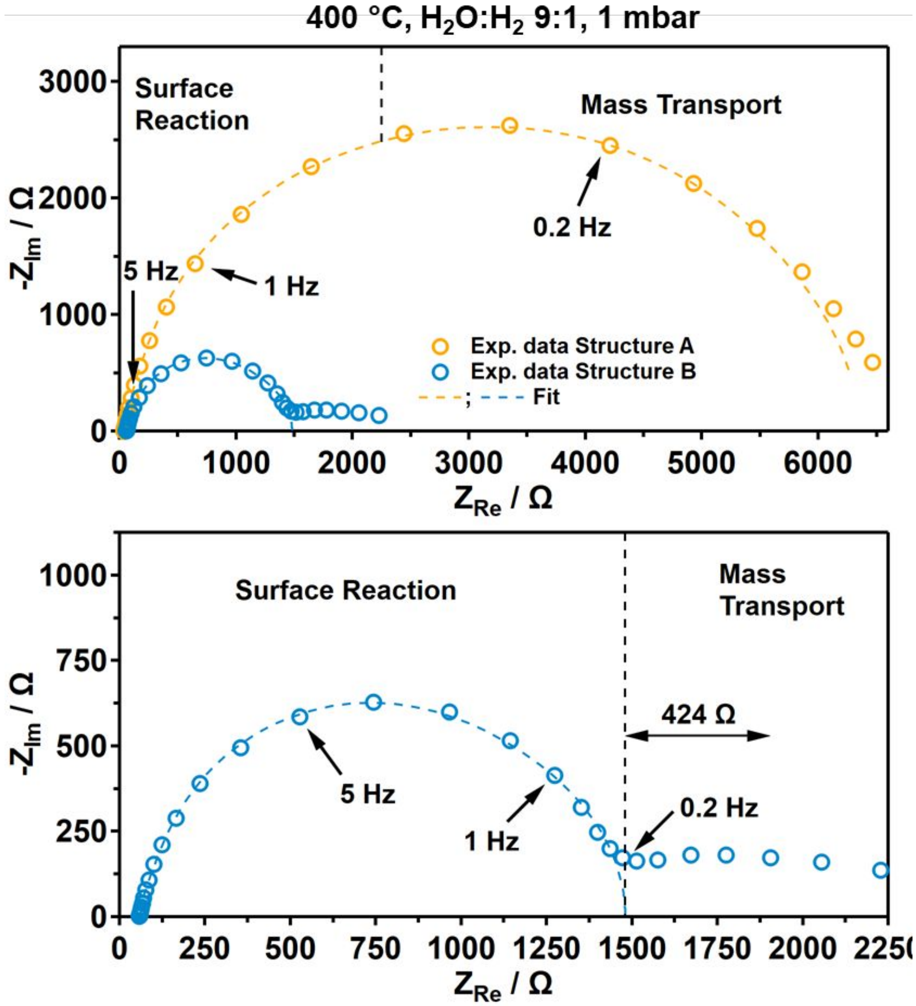


**Figure S6.** The EIS experimental data and fit of Structure A (orange) are compared to the respective data set of Structure B (blue). The EIS data were recorded at 400 °C in a 0.1 mbar $H_2$:$H_2O$ 1:9 mixture.

## Results of the EIS analysis

**Table S2.** Summary of identified (electro)chemical elementary processes and their frequencies at each temperature, including the subsequent comparison of the determined elementary process resistance *R* and capacitance *C* for each structure A and B. The change of these EIS parameters is expressed as "Ratio$_{A/B}$" and contrasted to the change of the structural parameters (A vs. B) and their ratios.

| T/°C | Process | f /Hz | $R_A$ /Ω<br>$C_A$ /µF | $R_B$ /Ω<br>$C_B$ /µF | Ratio$_{A/B}$ (EIS parameter) | Ratio (Structural parameter) | Structural parameter |
|---|---|---|---|---|---|---|---|
| 500 | Surface reaction | 5-30 | 196<br>207 | 165<br>31 | 1.2<br>**7** | -<br>- | |
| | Mass transport | <1 | 643[a]<br>75[b] | ≈24[c]<br>≈3[d] | - | 27 | TPB-length |

a) $R_{CT,MT}$ corrected by the CE mass transfer controlled CT resistance (660-17 = 643 Ω)
b) $Z_{MT}$ corrected by the CE mass transfer resistance (126-51 = 75 Ω)
c) calculated $R_{CT,MT}$ for Structure B (643/27 = 24 Ω)
d) calculated $Z_{MT}$ for Structure B (75/27 = 3 Ω)
e) total mass transport resistance of Structure B ($R_{CT,MT,WE} + Z_{MT,WE} + R_{CT,MT,CE} + Z_{MT,CE}$ = 24+3+17+51 = 95 Ω)

| T/°C | Process | f /Hz | $R_A$ /Ω<br>$C_A$ /µF | $R_B$ /Ω<br>$C_B$ /µF | Ratio$_{A/B}$ (EIS parameter) | Ratio (Structural parameter) | Structural parameter |
|---|---|---|---|---|---|---|---|
| 450 | Surface reaction | 2-8 | 850<br>110 | 705<br>27 | 1.2<br>4 | -<br>- | - |
| | Mass transport | <0.3 | 2893[a]<br>341[b] | ≈107[c]<br>≈214[d] | - | 27 | TPB-length |

a) $R_{CT,MT}$ corrected by the CE mass transfer controlled CT resistance (2920-25 = 2895 Ω)
b) $Z_{MT}$ corrected by the CE mass transfer resistance (417-76 = 341 Ω)
c) calculated $R_{CT,MT}$ for Structure B (2895/27 = 107 Ω)
d) calculated $Z_{MT}$ for Structure B (341/27 = 13 Ω)
e) total mass transport resistance of Structure B ($R_{CT,MT,WE} + Z_{MT,WE} + R_{CT,MT,CE} + Z_{MT,CE}$ = 107+13+25+76 = 221 Ω)

| T/°C | Process | f /Hz | $R_A$ /Ω<br>$C_A$ /µF | $R_B$ /Ω<br>$C_B$ /µF | Ratio$_{A/B}$ (EIS parameter) | Ratio (Structural parameter) | Structural parameter |
|---|---|---|---|---|---|---|---|
| 400 | Surface reaction | 2-4 | 1661<br>60 | 1379<br>26 | 1.2<br>2 | -<br>- | - |
| | Mass transport | <0.2 | 4284[a]<br>591[b] | ≈170[c]<br>≈424[d] | - | 27 | TPB-length |

a) $R_{CT,MT}$ corrected by the CE mass transfer controlled CT resistance (4380-96 = 4282 Ω)
b) $Z_{MT}$ corrected by the CE mass transfer resistance (745-154 = 591 Ω)
c) calculated $R_{CT,MT}$ for Structure B (4282/27 = 159 Ω)
d) calculated $Z_{MT}$ for Structure B (591/27 = 22 Ω)
e) total mass transport resistance of Structure B ($R_{CT,MT,WE} + Z_{MT,WE} + R_{CT,MT,CE} + Z_{MT,CE}$ = 159+22+96+154 = 431 Ω)

**Table S3.** Summary of the deconvolution of the elementary processes within the surface reaction pathway. The frequencies at each temperature, including the subsequent comparison of the determined elementary process resistance $R$ and capacitance $C$ for each structure A and B are provided. The change of these EIS parameters are expressed as "$Ratio_{A/B}$" and contrasted to the change of the structural parameters (A vs. B) and their ratios (cf. Table 3 and the "E-field modeling" section).

| T/°C | Process | f /Hz | $R_A$ /Ω<br>$C_A$ /µF | $R_B$ /Ω<br>$C_B$ /µF | $Ratio_{A/B}$ (EIS parameter) | Ratio (Structural parameter) | Structural parameter |
|---|---|---|---|---|---|---|---|
| 500 | Charge transfer | 15-30 | 16<br>707 | 152<br>31 | **0.11**<br>23 | **0.13**<br>8 | e-field energy |
| | Hydroxylation | 3 | 180<br>292 | 13<br>4380 | **14**<br>0.07 | **12**<br>0.08 | YSZ area |
| 450 | Charge transfer | 9 | 60<br>267 | 643<br>27 | **0.09**<br>10 | **0.13**<br>8 | e-field energy |
| | Hydroxylation | 1.5 | 790<br>187 | 62<br>1810 | **13**<br>0.10 | **12**<br>0.08 | YSZ area |
| 400 | Charge transfer | 5 | 132<br>228 | 1108<br>28 | **0.12**<br>8 | **0.13**<br>8 | e-field energy |
| | Hydroxylation | 1 | 3190<br>82 | 271<br>436 | **12**<br>0.19 | **12**<br>0.08 | YSZ area |

**Table S4.** Summary of the deconvolution of the elementary processes associated with $H_2$ desorption and CE exchange reactions and ohmic contributions from electrolyte and wiring. The frequencies at each temperature, including the subsequent comparison of the determined elementary process resistance *R* and capacitance *C* for each structure A and B are provided.

| **T / °C** | **Process** | **f / Hz** | **$R_A$ / Ω**<br>**$C_A$ / µF** | **$R_B$ / Ω**<br>**$C_B$ / F** |
|---|---|---|---|---|
| 500 | Electrolyte/Wiring | - | 20 | 14 |
| | Counter electrode exchange reaction | ≈700 | 3<br>88 | 2<br>77 |
| | Associative $H_2$ desorption | 80-110 | 5<br>376 | 16<br>95 |
| 450 | Electrolyte/Wiring | - | 24 | 27 |
| | Counter electrode exchange reaction | ≈600 | 3<br>70 | 4<br>59 |
| | Associative $H_2$ desorption | 50-90 | 14<br>128 | 33<br>93 |
| 400 | Electrolyte/Wiring | - | 47 | 57 |
| | Counter electrode exchange reaction | ≈300 | 6<br>97 | 5<br>99 |
| | Associative $H_2$ desorption | 35-65 | 26<br>167 | 39<br>66 |

**Table S5.** Summary of the deconvolution of the elementary processes shown in Figure 9b. The frequencies and elementary process resistance *R* and capacitance *C* for structure B under an applied polarization of -1.0 V are compared.

| **Process** | **f / Hz** | **$R_B$ / Ω** | **$C_B$ / µF** |
|---|---|---|---|
| Electrolyte/Wiring | - | 19 | - |
| Counter electrode exchange reaction 1 | $22 \cdot 10^3$ | 5 | 2 |
| Counter electrode exchange reaction 2 | $3 \cdot 10^3$ | 8 | 7 |
| Associative $H_2$ desorption | 450 | 14 | 24 |
| Charge transfer | 95 | 82 | 21 |
| Hydroxylation | 30 | 42 | 123 |
| Mass transport incl. CTR | 2 | 50 | - |

**References**

(1) Nečas, D.; Klapetek, P. Gwyddion: an open-source software for SPM data analysis. **2012**, *10* (1), 181-188. DOI: doi:10.2478/s11534-011-0096-2 (acccessed 2024-03-04).

(2) Fadley, C. S. Electron Spectroscopy: Theory, Techniques and Applications. In *Electron Spectrosc. Theory Tech. Appl. 2*, Baker, C. R. B. a. A. D. Ed.; Vol. 2; 1978.

(3) Jablonski, A.; Powell, C. *NIST Electron Effective-Attenuation-Length Database*; National Institute of Standards and Technology, Gaithersburg, MD, 2011.
